# Pairing cellular and synaptic dynamics into building blocks of rhythmic neural circuits. A tutorial

**DOI:** 10.3389/fnetp.2024.1397151

**Published:** 2024-06-25

**Authors:** James Scully, Jassem Bourahmah, David Bloom, Andrey L. Shilnikov

**Affiliations:** ^1^ Neuroscience Institute, Georgia State University, Atlanta, GA, United States; ^2^ TReNDS Center, Georgia State University, Atlanta, GA, United States; ^3^ Department of Mathematics and Statistics, Georgia State University, Atlanta, GA, United States

**Keywords:** locomotion, modeling, half-center oscillator, excitatory, inhibitory, rhythm generation, network physiology, neural networks

## Abstract

In this study we focus on two subnetworks common in the circuitry of swim central pattern generators (CPGs) in the sea slugs, *Melibe leonina* and *Dendronotus iris* and show that they are independently capable of stably producing emergent network bursting. This observation raises the question of whether the coordination of redundant bursting mechanisms plays a role in the generation of rhythm and its regulation in the given swim CPGs. To address this question, we investigate two pairwise rhythm-generating networks and examine the properties of their fundamental components: cellular and synaptic, which are crucial for proper network assembly and its stable function. We perform a slow-fast decomposition analysis of cellular dynamics and highlight its significant bifurcations occurring in isolated and coupled neurons. A novel model for slow synapses with high filtering efficiency and temporal delay is also introduced and examined. Our findings demonstrate the existence of two modes of oscillation in bicellular rhythm-generating networks with network hysteresis: i) a half-center oscillator and ii) an excitatory-inhibitory pair. These 2-cell networks offer potential as common building blocks combined in modular organization of larger neural circuits preserving robust network hysteresis.

## 1 Introduction

Animal locomotion is facilitated and determined by small neural circuits, known as central pattern generators (CPGs) (Selverston, 1985; Marder and Calabrese, 1996; Katz et al., 2007; Calabrese et al., 2016; Katz, 2016; Marder et al., 2022) that can autonomously produce rhythmic patterns of neural activity. Despite their simplicity, the coordination and control of these CPGs can exhibit remarkable complexity. Studying animal locomotion offers a valuable opportunity to comprehend the intricate relationships between network components that lead to adaptive behavior. The current paper is focused on our recent collaborative efforts to model stable swim rhythmic patter of two species of the sea slugs *Melibe leonina* (Watson et al., 2002; Watson and Newcomb, 2002; Thompson and Watson, 2005) and *Dendronotus iris* (Sakurai et al., 2011). The CPG circuits underlying their behaviors have been studied extensively in both species (Newcomb et al., 2012; Sakurai et al., 2014; Sakurai and Katz, 2015; Sakurai and Katz, 2016). All neurons in the CPGs have been identified, and their synaptic connections have been determined with careful pairwise electrophysiological recordings (Sakurai and Katz, 2017; Sakurai and Katz, 2019; Sakurai and Katz, 2022). The swim CPGs in these sea slugs do not include endogenously bursting pacemakers. Hence, each circuit should be viewed as a whole rather than by looking at specific pacemaker cells. The working hypothesis is that the given CPG circuits function at controlled oscillatory states emerge largely through the synergetic interactions among the coupled components with similar dynamic and nonlinear properties. This is the main driver and the starting point of our computational study, which is focused on identifying what cellular and synaptic qualities can warrant robust generation of slow oscillations in two specific bicellular blocks, which happened to be symmetrically built into the swim CPG circuits of the given sea slugs. Despite the absence of autonomous bursting in individual cells, the circuit can be decomposed into sub-networks that possess the capacity to exhibit rhythmic bursting. Our modeling efforts have led us to investigate how the coordination of these fundamental network oscillators results in flexible and adaptive rhythmic patterns. The purpose of this paper is to highlight the properties of two such oscillators, each with distinct mechanisms for generating emergent bursting. These models have been directly derived from our collaborative modeling and experimental research on *Melibe* and *Dendronotus* swim CPGs.

The CPGs in animal locomotion can be broadly categorized into two categories: those driven by pacemaking cells, (Selverston et al., 1976; Marder, 1994; Stein et al., 1999; Prinz et al., 2004; Goaillard et al., 2009; Selverston, 2013), and those that generate rhythmic patterns through network-level mechanisms as in the case of the given two sea slugs. This study focuses on the latter type of CPGs, where slow oscillations emerge from the reciprocal interaction of multiple cells in the network, rather than from the oscillatory activity produced by a single pacemaking cell. A challenge arises, as the distinction between bursting and non-bursting cells becomes blurred under the influence of synaptic inputs (Alaçam and Shilnikov, 2015). To address this critical issue, we ensure that hysteresis (which is a prerequisite for endogenous bursting in cell models) between the tonic spiking manifold and the stable quiescent manifold is eliminated, regardless of synaptic drive. In the context of this paper the term network hysteresis refers to a scenario when the overlap emerges temporarily a post-synaptic interneuron due to one-way inhibition caused by a pre-synaptic one. The literature on small neural networks has documented several mechanisms underlying bursting behavior, including post-inhibitory rebound (PIR), escape, and release (Perkel and Mulloney, 1974; Ermentrout and Kopell, 1986; Wang and Rinzel, 1992; Skinner et al., 1994; Baer et al., 1995; Sharp et al., 1996; Skinner et al., 1999; Kopell and Ermentrout, 2002; Angstadt et al., 2005; Matveev et al., 2007; Shilnikov et al., 2008; Daun et al., 2009; Szucs et al., 2009; Jalil et al., 2010; Shilnikov, 2012b; Nagornov et al., 2016). PIR occurs when a neuron is rapidly depolarized after being freed from inhibition, resulting in a spike train. Escape occurs when a hyperpolarized neuron begins firing, followed by inhibition that terminates its spike train. Release occurs when a spike train ends, removing inhibition and allowing a previously silent neuron to burst. The potential for all three mechanisms exist in the cells which constitute the CPGs in *Dendronotus* and *melibe* (Sakurai and Katz, 2022). However, these mechanisms do not fully explain the oscillations observed in the swim CPGs of *Melibe* and *Dendronotus*, as the circuits may contain a redundancy of all three mechanisms.

In this study we examine two well-known types of neural circuits composed of two cells, which will be incorporated into complete CPG models in future research. The first circuit is a variation of a half-center oscillator (HCO), which was initially described in the seminal study by T.G. Brown (Brown, 1911). This HCO consists of two non-bursting neurons that are reciprocally connected by inhibitory synapses, resulting in alternating bursting patterns. Unlike HCOs that rely on hysteresis pre-existing in individual models, the HCO in this study comprises of non-spiking cells, providing flexibility and adaptability. Our findings demonstrate that the emergent bursting mechanism is the same, regardless of whether the cells are tonic spikers or quiescent.

The second pair-wise network under examination is an asymmetric circuit composed of an excitatory and an inhibitory neuron. Excitatory/inhibitory (E/I) oscillatory networks have been previously explored, particularly as population models (Wilson and Cowan, 1972). However, mollusk CPG networks consist of a limited number of cells and hence such population models are well suited for their in-detail investigation. In this study, we present a 2-cell E/I network modeled using the Hodgkin-Huxley formalism. Previous research on neurological systems featuring oscillations and E/I interactions include neural mass models of gamma rhythm generation (Börgers et al., 2005; Tiesinga, 2009; Buzsáki and Wang, 2012), spindle oscillations (Golomb et al., 1994), working memory (Brunel and Wang, 2001), and respiratory rhythms in the pre-Bötzinger complex (Rubin, 2006), where the concept of emergent bursting was investigated in such large ensembles.

**FIGURE 1:**
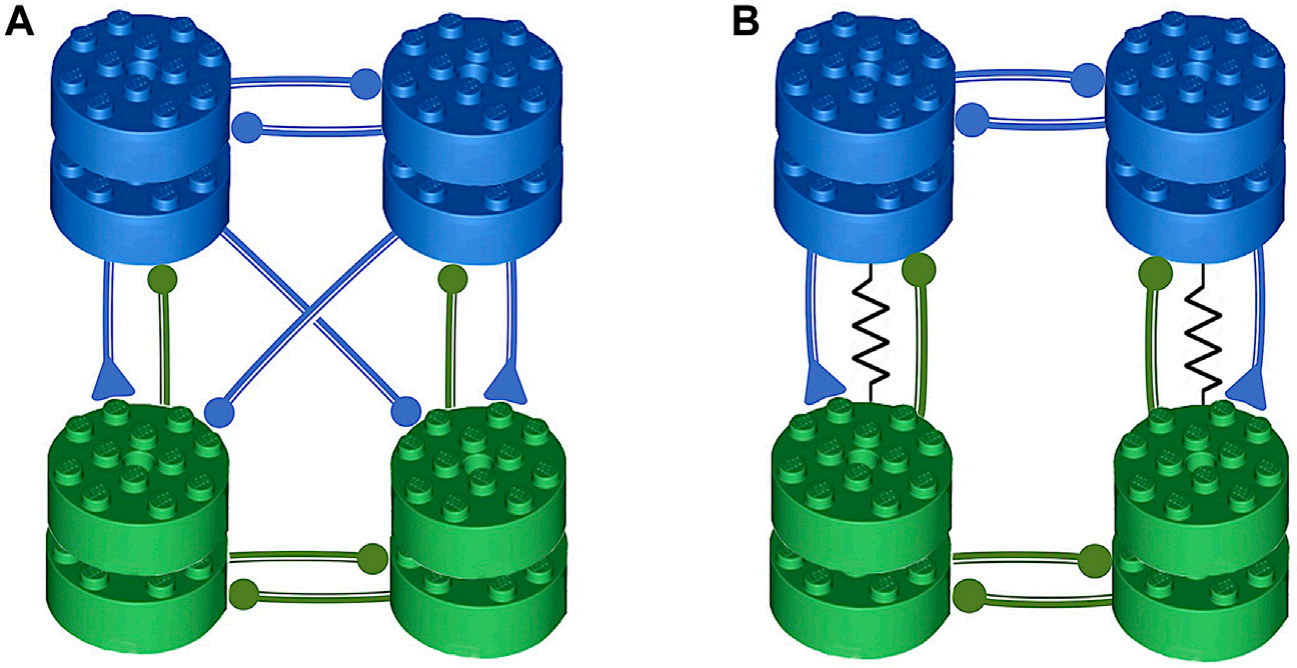
The illustration of the symmetric, pair-wise organization of the swimming central pattern generators (CPGs) in the sea slug species: *Melibe leonina* in panel **(A)**, and *Dendronotus iris* in panel **(B)**. The assembly of these CPGs revolves around two fundamental motifs–a half-center oscillator (HCO) made of two reciprocally inhibitory neurons, and an excitatory-inhibitory (E/I) module. These subnetworks work in coordination to establish oscillatory motor circuits. Components belonging to the HCO are represented with the same color, whereas the E/I components are arranged in vertical columns. Symbols are used to depict synaptic activity, with circles symbolizing inhibitory and triangles representing excitatory action, both on the postsynaptic side of the synapse.

In this study, we present models for two 2-cell circuits: the half-center oscillator (HCO) and excitatory/inhibitory (E/I) modules that are designed to exhibit robust oscillation mechanisms, devoid of latent bursting. These modules serve as building blocks for the larger swim CPG models in the sea slugs *Melibe leonina* and *Dendronotus iris*. The schematic depiction of the swim CPG circuitry in these sea slugs, as schematically shown in Figure 1, illustrates our assertion that the HCO and E/I-module components are designed to promote and sustain stable slow rhythms at the network level, that are resilient to changes in cellular and synaptic parameters, as well as intrinsic and external perturbations.

## 2 Methods and models

### 2.1 Description of the swim interneuron (SiN) model

Our objective in choosing a neuron model was its biological plausibility, even though cellular currents in swim CPG interneurons of the sea slugs *Melibe leonina* and *Dendronotus iris* have not been identified or specified. As our starting point for a conductance-based model to describe the swim CPG interneurons, we picked the original Plant model (Plant and Kim, 1975; Plant and Kim, 1976; Plant, 1981) of the endogenously bursting cell R15 in the sea slug *Aplysia californica* (Rittenhouse and Price, 1985; Levitan and Levitan, 1988) mainly because it was a well characterized model with variable spike frequency (Rinzel, 1985; Rinzel, 1987). An accurate mathematical analysis of the Plant model was initially done in Refs (Rinzel et al., 1986; Rinzel and Lee, 1987). using slow-fast dissection, while a variety of dynamical properties of the R15-neuron models were examined later in following publications, see Refs (Canavier et al., 1991; Bertran, 1993; Canavier et al., 1993; Butera et al., 1995; Butera, 1998; Sieling and Butera, 2011; Alaçam and Shilnikov, 2015) and references therein.

Both the model itself and the method of analysis used throughout this paper differ from the original Plant burster. We emphasize that the swim interneurons in both sea slug CPGs have *not* been observed to burst endogenously (Sakurai et al., 2011; Newcomb et al., 2012; Sakurai et al., 2014; Sakurai and Katz, 2015; Sakurai and Katz, 2016; Sakurai and Katz, 2017; Sakurai and Katz, 2019; Sakurai and Katz, 2022). Moreover, these interneurons are not latent bursters either, as they do not burst even when perturbed with constant external currents (Alaçam and Shilnikov, 2015). Accurate neurophysiological experiments on the swim CPGs demonstrated that their interneurons are either quiescent at most times, or become tonic-spiking during swim episodes when receiving excitatory drives from sensory cells. This strongly suggests that the slow bursting (of period ranging from 2 through 14 s, resp., in juvenile and grown animals) observed in the experimental studies on the swim CPGs in the sea slugs is indeed a *network-level* dynamical phenomenon emerging due to nonlinear interactions between the interneurons orchestrated by complex coordination of fast and slow currents, including synaptic ones. In our modeling efforts, it is important to address the observed activity of the CPG interneurons in normal function and under perturbation, as well as describe the realistic timescales of various synapses in order to explore the network-level rhythmogenesis in the given CPG-circuits.

Following Refs (Shilnikov and Cymbalyuk, 2004; Shilnikov, 2012a; Alaçam and Shilnikov, 2015). we introduce two additional parameters, Δ_Ca_ and Δ_*x*_, to manipulate and eliminate bursting from the swim interneuron (SiN) model. We also add an *h*-current to prevent excessive hyperpolarization. We use an averaging approach for fast-slow decomposition as opposed to the original work. The reader will find more details on the averaging approach, including the notion of average nullclines to locate a periodic orbit in the phase space and corresponding to tonic-spiking activity in the proposed SiB model in Appendix I in the Supplementary Material below.

The original Plant model includes the following fast currents: the inward sodium and calcium (*I*_*I*_), the outward potassium (*I*_*K*_), necessary for the spike generation, a depolarizing h-current (*I*_*h*_), along with the generic ohmic leak (*I*_*leak*_) current. The gradual spike frequency adaptation and post-inhibitory rebound in the model are due to the slow dynamics of two currents: the TTX-resistant inward sodium and calcium current (*I*_T_) and outward calcium-sensitive potassium current (*I*_*KCa*_).

$$C_{m}V^{\prime }=-I_{I}-I_{K}-I_{\mathrm{leak}}-I_{h}-I_{T}-I_{\mathrm{KCa}},\;$$ (1)

$$h^{\prime }=\frac{h_{\infty }\left(V\right)-h}{\tau_{h}\left(V\right)},\;n^{\prime }=\frac{n_{\infty }\left(V\right)-n}{\tau_{n}\left(V\right)},$$ (2)

$$y^{\prime }=\frac{1}{2}\left[\frac{1}{1+e^{10\left(V-50\right)}}-y\right]/\left[7.1+\frac{10.4}{1+e^{\left(V+68\right)/2.2}}\right]$$ (3)

with the membrane capacitance *C*_*m*_ = 1. Here the dynamic variables are the membrane voltage *V*(*t*), the gating probabilities *h*(*t*), *n*(*t*), and *y*(*t*). This ODE system describing the R15 Plant burster includes a fast subsystem to generate repetitive tonic-spiking or quiescent activity, depending on the level of drive from its slow subsystem. This spike-generating machinery of the fast subsystem consists of the four aforementioned currents, which are governed by the following equations:

$$I_{I}=g_{I}\;h\;m_{\infty }^{3}\left(V\right)\;\left(V-E_{I}\right),$$ (4)

$$I_{K}=g_{K}\;n^{4}\;\left(V-E_{K}\right),$$ (5)

$$I_{h}=g_{h}\;\frac{y\;\left(V-E_{h}\right)}{{\left(1+e^{-\left(V-63\right)/7.8}\right)}^{3}},$$ (6)

$$I_{\mathrm{leak}}=g_{L}\left(V-E_{L}\right)$$ (7)

Here, the activation of the inward sodium current is assumed instantaneous and therefore described by an analytical (sigmoidal) equation $m_{\infty }^{3}\left(V\right)$ , rather than a corresponding fast ODE. The third current describes a depolarizing *h*-current that activates as the voltage drops down below −50mV, see Eq. 3 above.^1^

Recurrent alternation between fast spike trains and slow quiescent episodes in the endogenous Plant burster is reciprocally modulated by the slow inward TTX-resistant Na^+^-Ca^2+^ current and slow outward Ca^2+^ activated *K* ^+^ given, respectively, by

$$I_{T}=g_{T}x\left(V-E_{I}\right),$$ (8)

$$I_{\mathrm{KCa}}=g_{\mathrm{KCa}}\frac{\left[Ca\right]}{0.5+\left[Ca\right]}\left(V-E_{K}\right)$$ (9)

with two dynamical variables: the calcium concentration [Ca(*t*)] and a voltage gated probability *x*(*t*), which are governed by following coupled slow system:

$$x^{\prime }=\frac{1}{\tau_{x}}\left[\frac{1}{1+e^{-0.15\left(V+50-\Delta_{x}\right)}}-x\right],\tau_{x}\gg 1,\;$$ (10)

$${\left[Ca\right]}^{\prime }=\rho \left(K_{c}x\left(E_{Ca}-V+\Delta_{\text{Ca}}\right)-\left[Ca\right]\right),\rho \ll 1,$$ (11)

where Δ_*x*_ and Δ_Ca_ are bifurcation parameters introduced to control slow dynamics of the SiN-model; we will discuss their action below. The reader can find the detailed description of biophysical parameters in Appendix II in the Supplementary Material below.

**FIGURE 2:**
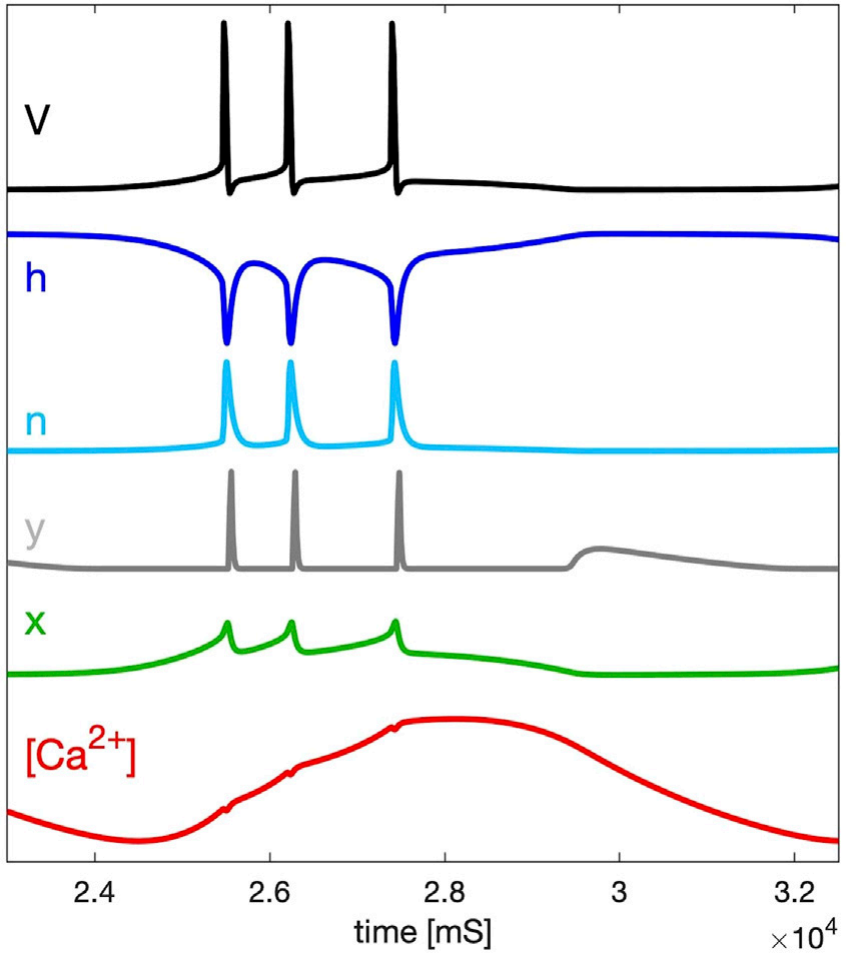
Time scales of the fast variables of the SiN-model: membrane voltage *V*, and gating probabilities *h*, *n*, and *y* (within the [0–1] range) for the inward *Na* ^+^ sodium and Ca^2+^ calcium, *K* ^+^ potassium, and *h*-currents, resp., compared against the slow variables, *x* and [Ca^2+^], introduced in both calcium-coupled currents.

The time scales of the state variables: *V*(*t*), probabilities *h*(*t*), *n*(*t*), *y*(*t*) and *x*(*t*) and calcium concentration [Ca](*t*) that gate the indicated cellular currents in the model are presented in Figure 2. Note that depending on the chosen value of the time constant *τ*_*x*_, the average rate of change of the gating *x*-variable can be altered: it can be visually as fast (at *τ*_*x*_ = 100 s^−1^) as that of the fast subsystem, or can be slowed down to match the rate of change of the [Ca]-variable at larger values such as *τ*_*x*_ = 273 s^−1^ also used in this study. Nonetheless, the *x*-dynamics is included in the slow subsystem for a few reasons. First, for the sake of historical continuity, *x*(*t*) was treated as a slow variable in the original slow-fast dissection analysis of this model proposed in Ref. (Rinzel and Lee, 1987). Second, the *x*-dynamics does not contribute (but regulate) to the spike generation, which is solely due to the fast sodium-calcium and potassium currents. Instead its reciprocal nonlinear interaction with the [Ca]-dynamics is the key factor that provides the Plant model with a slow hysteresis necessary for the onset of endogenous bursting composed of alternating fast spike trains and quiescent episodes.

The Δ_*x*_-parameter represents a deviation from the voltage value −50mV at which the TTX-resistant Na^+^-Ca^2+^ current is half-activated, see Eq. 10 and the corresponding activating function $x_{\infty }\left(V\right)=1/\left(1+e^{-0.15\left(V+50-\Delta_{x}\right)}\right)=1/2$ . The second parameter Δ_Ca_ introduced in Eq. 11 shifts the calcium reversal potential from a hypothetically high value +140mV. This voltage is too high to be measured experimentally, so changing the reversal potential is biologically plausible.

**FIGURE 3:**
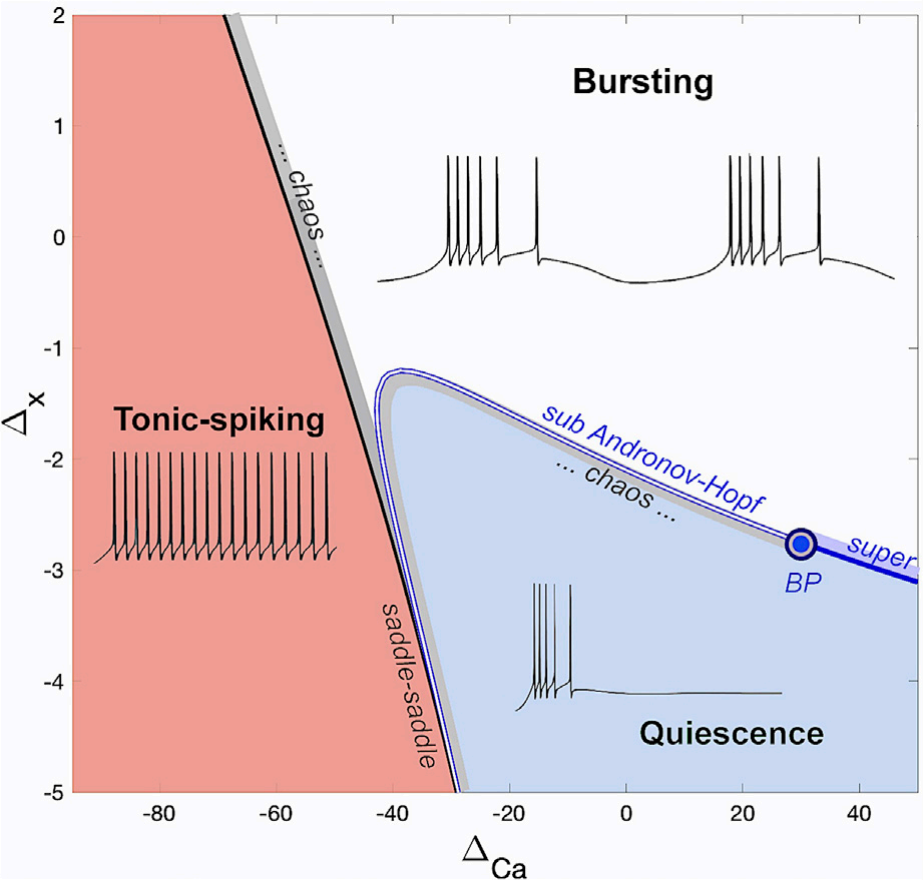
The (Δ_Ca_, Δ_*x*_)-bifurcation diagram of the adapted SiN-model with the three regions corresponding to tonic-spiking, bursting, and quiescent activity.

The bifurcation diagram in Figure 3 shows the different regions of activity in the SiN-model based on the parameter pairs $\left(\Delta_{\text{Ca}},\;\Delta_{x}\right)$ . The diagram is divided into three main regions: tonic-spiking, bursting, and hyperpolarized quiescent. The borderlines between these regions highlight the parameter dependence of the neural activity in the model. There are two main transition routes from one region to another: from tonic-spiking to bursting and from quiescence to bursting. The first route begins with a saddle-saddle bifurcation (Shilnikov, 1963; Shilnikov et al., 1998; Afraimovich et al., 2014; Afraimovich et al., 2014; Afraimovich et al., 2017; Gonchenko et al., 2022) and is followed by a narrow region of transitional chaos. The second route passes through the Andronov-Hopf (AH) bifurcation curve and has a codimension-two Bautin (Andronov and Leontovich, 1937; Andronov et al., 1938; Andronov et al., 1967) point (BP)^2^ on it. In-depth analysis of the origin and nature of chaos near these transition will be given in our forthcoming paper soon to be submitted. On the left side of this point, the bifurcation is sub-critical, leading to chaotic bursting within a transition layer. On the right side, it is super-critical, giving rise to small sub-threshold oscillations that eventually morph into large bursting. The SiN-model is only capable of demonstrating tonic-spiking activity or quiescence below the level Δ_*x*_ = −3.5mV, which is therefore the region of interest for network oscillations where cells do not burst endogenously. The proposed SiN-model was calibrated so that it can only show tonic-spiking or quiescent activity below Δ_*x*_ = −3.5mV. This particular range constitutes the region of interest with regards to network oscillations as it is characterized by the absence of endogenous bursting in the individual interneurons.

### 2.2 Bifurcation analysis with the Δ_*x*_ and Δ_Ca_-parameters

In the transition between tonic-spiking and bursting activity, a series of nonlocal bifurcations occur as the Δ_Ca_-parameter is increased (at some fixed Δ_*x*_ = 0). This process includes a period-doubling cascade that leads to chaotic dynamics within a narrow region in the parameter plane. In this region, the round periodic orbits for tonic-spiking activity change into two time-scale bursting orbits consisting of fast spike trains and slow quiescent phases. This region is adjacent to a local saddle-saddle bifurcation curve (black line). Unlike a saddle-node bifurcation that results in the merging and vanishing of saddle and stable equilibrium states, a saddle-saddle bifurcation results in the emergence of two saddles of opposite topological types nearby in 3D and higher dimensional systems (Shilnikov et al., 1998; Afraimovich et al., 2014; Afraimovich et al., 2017).

In the swim interneuron model, the transition between hyperpolarized quiescence and bursting as the Δ_*x*_-parameter is increased (for fixed Δ_Ca_) involves a series of consecutive bifurcations. A local AH bifurcation occurs through which a stable equilibrium state representing the neural quiescence becomes unstable. The criticality of this bifurcation can be sub- or super-critical, and is determined by the sign, positive or negative, of the Lyapunov coefficient at the bifurcation.

A bottom-up route to bursting on the right from the BP-point where the AH bifurcation is a supercritical one, results in the gradual onset of stable sub-threshold oscillations that eventually transform into fully developed bursting at larger Δ_*x*_-values. On the left from the BP, the route to bursting is more complicated and can result in complex bursting oscillations, chaotic sub-threshold oscillations, unpredictably varying trains of spikes, or a combination of these. Additionally, bistability can occur in the SiN-model as a result of bursting co-existing with a stable quiescent state. A detailed bifurcation analysis of the transitions between neural activity types will be carried out in a future study.

### 2.3 Phase space dissection of the swim interneuron burster

The following four curves in the ([Ca], *x*)-phase plane serve to interpret the dynamics of the swim interneuron model and its transformations. 1) The *x*-nullcline represents the set of points where *x* does not change. 2) The [Ca]-nullcline is the set of points where the slow variable [Ca] is unchanged. 3) The average ⟨*x*⟩ nullcline represents the set of points where *x* does not change when averaged over the spiking periodic orbit. 4) The saddle node on the invariant circle (SNIC) curve^3^ is a bifurcation curve that illustrates the boundary between spiking and quiescence in the fast subsystem. Understanding the interplay between these curves is crucial in understanding the overall dynamics of the SiN-model.

**FIGURE 4:**
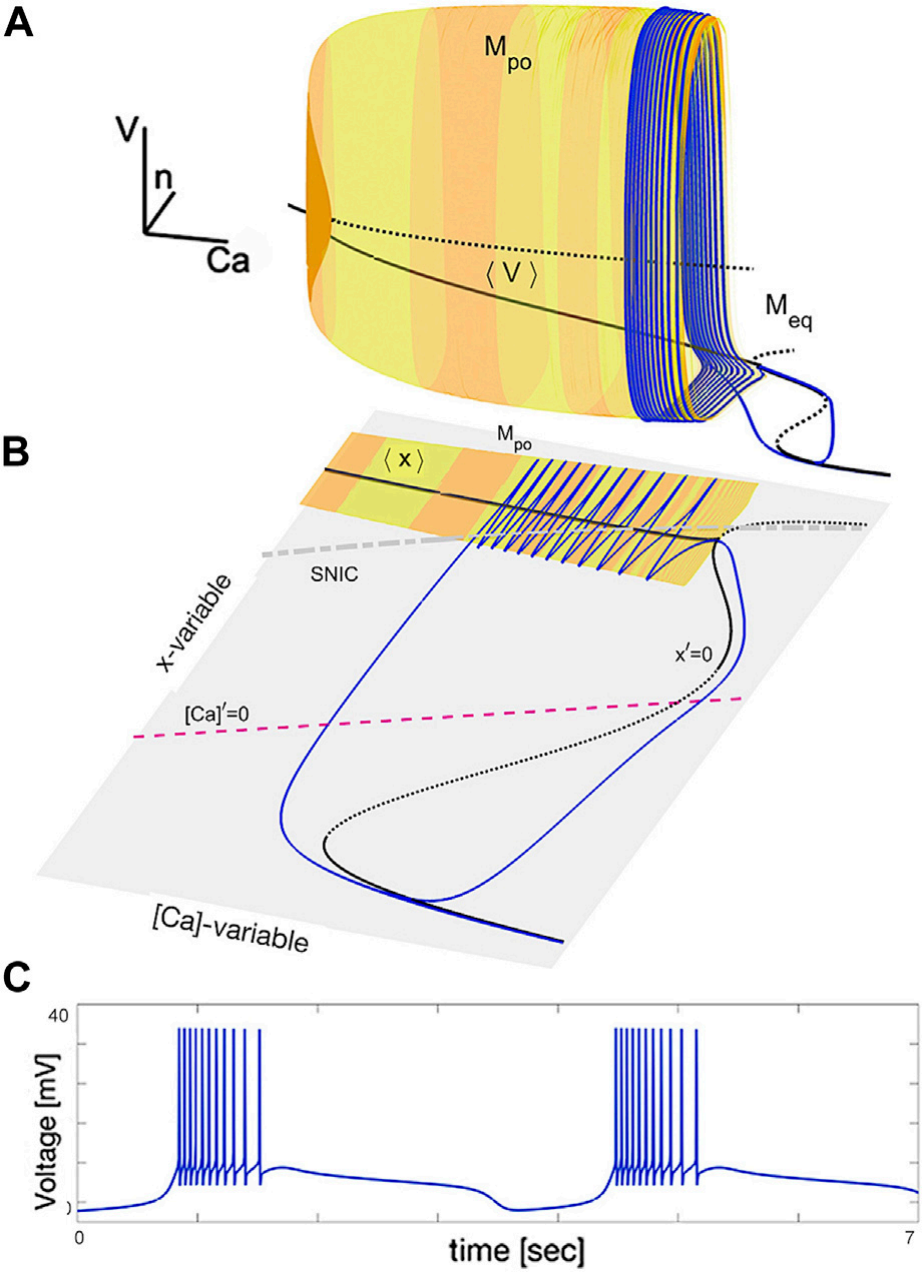
Phase subspace of the adapted SiN-model at specific parameter values. Panel **(A)** shows the critical manifold *M*_po_ made up of tonic-spiking periodic orbits and the curve ⟨*V*⟩ representing the time averages of fast oscillations. The blue trajectory, turning around the *M*_po_-surface and sliding along the *M*_eq_-curve, is a bursting periodic orbit. Panel **(B)** shows the slow dynamics of the SiN-model in the ([Ca], *x*)-subspace, with two stable overlapping branches of the average nullcline ⟨*x*⟩ and the x-nullcline *x*′=0 representing the ⟨*V*⟩- and *M*_eq_ manifolds. Panel **(C)** displays the voltage trace of the bursting orbit with alternating episodes of hyperpolarized quiescent transients and fast spikes.

The illustration in Figure 4 provides a visual representation of the dynamics in the swim interneuron burster. Figure 4A shows a 3D subspace ([Ca], *n*, *V*) and depicts a pair of critical manifolds: a 2D cylinder-shaped surface *M*_po_ consisting of periodic orbits and a 1D multi-folded space curve *M*_eq_ made up of equilibria. Figure 4B depicts the slow ([Ca], *x*)-phase plane and shows the interplay between the *x*-nullcline, [Ca]-nullcline, the average ⟨*x*⟩ nullcline, and the SNIC-curve, which marks the transition between the active and silent phases of the fast subsystem. The SNIC-curve separates the oscillatory, tonic-spiking activity (above the SNIC-curve) from the hyperpolarized quiescent phase (below the SNIC-curve). The periodic orbits and equilibria are found through numerical continuation in the fast subsystem, and the bursting periodic orbit is shown as a blue line on the surface *M*_po_. The voltage trace of this periodic orbit is displayed in Figure 4C.

The solid black curve labeled by ⟨*V*⟩ in Figure 4A and its equivalent denoted by ⟨*x*⟩ in 4B represent the “center of gravity” of the critical manifold *M*_po_. This manifold *M*_po_ is comprised of periodic orbits representing the tonic-spiking activity in the SiN-model. The curve is found by averaging the fast coordinates, such as *V*_po_(*t*) and *x*_po_(*t*), of each periodic orbit over its period using the formula:

$$\langle V\rangle =\frac{1}{T}\int_{0}^{T}V_{\text{po}}\left(t\right)dt\;\text{and}\;\langle x\rangle =\frac{1}{T}\int_{0}^{T}x_{\text{po}}\left(t\right)dt,$$ (12)

This curve starts at the point where *M*_po_ collapses into the *M*_eq_-manifold and ends at the top fold on the 1D manifold, along which the bent SNIC-curve passes.

The interplay between these nullclines determines the overall behavior of the slow subsystem. The intersection of the *x*- and [Ca]-nullclines is a fixed point of the slow subsystem, representing an equilibrium solution, and its stability is determined by the direction of the vector field at that point. If the vector field points towards the fixed point, then it is stable, otherwise it is unstable. Moreover, the *x*-nullcline also marks the transition between the active and silent phases of the fast subsystem as discussed previously, as it corresponds to the 1D manifold *M*_eq_ in the full phase space.

In the ([Ca], *x*)-plane, one can observe a bifurcation line marking the transition between the active and silent phases of the fast subsystem, called the saddle node on an invariant circle (SNIC). The SNIC-curve indicates the threshold value of [Ca] and *x* below which the fast subsystem is hyperpolarized and quiescent, and above which it is tonic-spiking.

In summary, the position and stability of the fixed points, the direction of the vector field, and the SNIC-curve all determine the overall behavior of the slow subsystem in the ([Ca], *x*)-phase plane, which in turn affects the overall dynamics of the swim interneuron model.

The geometric analysis of the nullclines helps understand the slow dynamics of the SiN-model. The intersection of the [Ca] and *x*-nullclines in the ([Ca], *x*)-plane represents an equilibrium state of the slow subsystem. Whether this state is stable or unstable depends on the intersection of the [Ca]-nullcline with the stable (solid) or unstable (dotted) branch of the *x*-nullcline. The tangency between the nearly straight [Ca]-nullcline and the bending Σ-shaped *x*-nullcline corresponds to a saddle-node or saddle-saddle bifurcation of the equilibrium states in the model, as seen in the bifurcation diagram. The transverse crossing of the [Ca]-nullcline through a knee of the *x*-nullcline represents an AH bifurcation in the slow subsystem of the SiN-model.

The behavior of the full model is determined by the geometric configuration of the slow nullclines in the ([Ca], *x*)-plane. A stable equilibrium in both slow and fast subspaces is required for attractor behavior. Only stable sections of the slow-motion or critical manifolds, tonic-spiking *M*_eq_ and quiescent *M*_po_, can correspond to observable spike generation and resting states in the SiN-model. Therefore, there are three options to interpret types of neural activity exhibited by the given model based on dynamics in its slow compartment: i) a stable equilibrium state located below the SNIC-curve in ([Ca], *x*)-plane is also a steady state in the whole system and corresponds to hyperpolarized quiescence in the SiN-model; ii) a stable equilibrium located above the SNIC-curve in the ([Ca], *x*)-plane is a stable periodic orbit in the phase space and corresponds to tonic-spiking activity; or iii) any unstable, repelling or saddle, equilibrium state located below the SNIC-curve corresponds to a saddle one on the phase space of the SiN-model, and is hence invisible in general, as we said above. This is not the case in critical situations typically occurring at complex, chaotic transitions between activity types where saddle orbits become inflectional and determine bifurcation routes. Furthermore, roles of saddles are imperative when the system under consideration is bistable or multistable, as they determine boundaries between basins of co-existing attractors, including quiescent, tonic-spiking and/or various bursting ones (Shilnikov and Cymbalyuk, 2004; Shilnikov et al., 2005a; Cymbalyuk and Shilnikov, 2005; Shilnikov and Kolomiets, 2008; Shilnikov, 2012a; Ju et al., 2018).

The geometric configuration based on the average ⟨*x*⟩ nullcline connecting to the equilibrium nullcline *x*′ = 0 in the ([Ca], *x*)-phase plane is reminiscent of the single cubic nullcline found in a relaxation oscillator and its biological interpretation–the Fitzhugh–Nagumo neuron. The hysteresis in this construction, marked by two overlapping branches, is a necessary condition for oscillations in the Fitzhugh–Nagumo neuron, which would correspond to endogenously bursting oscillations in the SiN-model. However, this is not sufficient for oscillations. To ensure oscillations the overlapping branches may not intersect with the other nullcline. Figure 4B depicts such a configuration where the nullcline [Ca]′ = 0 crosses the nullcline *x*′ = 0 throughout its unstable branch. This results in the onset of a stable limit cycle (blue line) in the ([Ca], *x*)-phase plane, which corresponds to a bursting periodic orbit and shown in Panel A where the orbit turns around the tonic-spiking manifold *M*_po_, slides onto the quiescent manifold *M*_eq_, and back to *M*_po_ and so forth to generate the voltage trace in Panel C of the same figure.

### 2.4 Transitions due to intrinsic and external parameters

**FIGURE 5:**
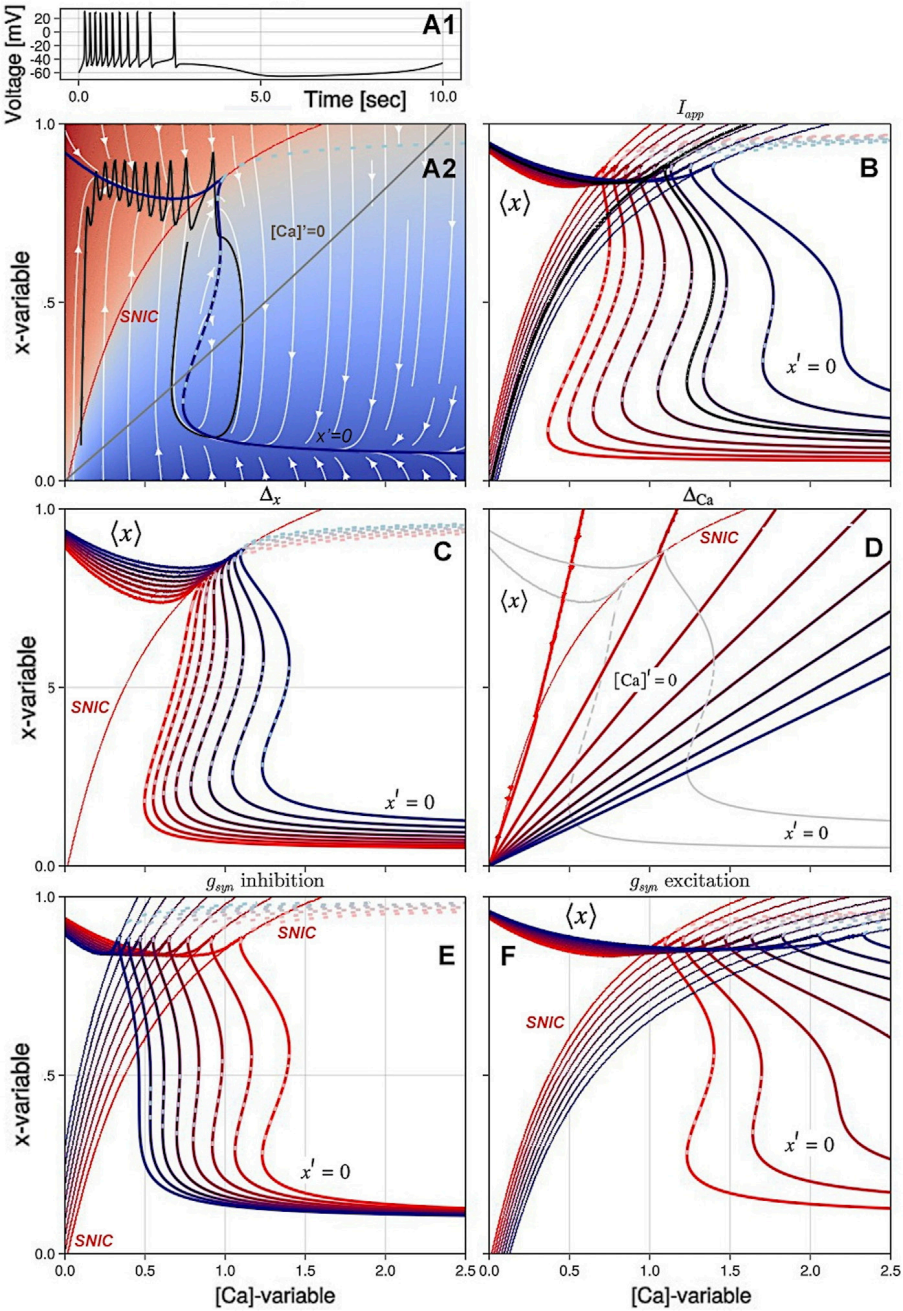
Variations of the principal control parameters in the SiN-model lead to changes in the slow ([Ca], *x*) phase plane and result in different types of activity in the voltage trace **(A1)**. In Panel **(A** **2** **)**, the blue line is the average nullcline ⟨*x*⟩ and the solid/dotted dark blue line is the stable/unstable branch of the x-nullcline *x*′=0 representing the ⟨*V*⟩- and *M*_eq_ manifolds, respectively. The grey line is the nullcline [Ca]′=0, and the red line is the SNIC bifurcation curve. The background color shows the average low or high voltages in the plane. In Panel **(B)**, the hysteresis in the slow dynamics is shown as the external current *I*_*ext*_ changes from −0.1 (red lines) to 0.05 (blue lines). Panel **(C)** shows the effect of decreasing the Δ_*x*_ parameter from 0 to −4 on the overlap of the nullcline *x*′=0 and the average nullcline ⟨*x*⟩. In Panel **(D)**, increasing Δ_Ca_ from −100 to 350 shifts the intersection of the nullclines [Ca]′=0 and *x*′=0 across the SNIC curve and changes the type of activity in the voltage trace from tonic-spiking to quiescence or bursting. In Panels **(E, F)**, changes in the maximal conductance *g*_*syn*_ of inhibitory and excitatory currents result in rearrangements of the nullclines and the stability of the x-nullcline *x*′=0.

External parameters are used to control the activity of the neuron in an isolated environment. The constant applied current, *I*_*app*_, allows for a direct control over the neuron’s voltage and changes the voltage trajectory in the phase plane. The synaptic drive or current, *I*_*syn*_, represents the interaction between neurons and the exchange of information through synaptic transmission. This interaction can either excite or inhibit the neuron based on the value of the reversal potential, *E*_*rev*_. The panels of Figure 5 demonstrate the overall effects of individual variations of four control parameters *I*_*app*_, Δ_*x*_, Δ_Ca_, and *g*_*syn*_ on the geometric organization and its rearrangements of the slow ([Ca], *x*)-phase plane.

The bifurcation diagram in Figure 3 shows that as the intrinsic parameter Δ_*x*_ is decreased, the SiN-model morphs from an endogenous burster to a no-burster. This rearranges the ([Ca], *x*)-phase plane, causing the overlapping of nullclines for periodic orbits and equilibria to break down. This is shown in Figure 5C, where a decrease in Δ_*x*_ makes the nullcline *x*′ = 0 bend, leading to the emergence of an upper stable branch below the SNIC-curve, and shifts the Σ-shaped nullcline *x*′ = 0 to the right, eliminating the overlap with ⟨*x*⟩. This suggests that at Δ = −4mV, the model transitions directly from the tonic-spiking activity to the hyperpolarized quiescent state as Δ_Ca_ is varied.

Figure 5D shows how variations in Δ_Ca_ affect the shape and intersection of the [Ca]-nullcline and the *x*′ = 0 nullcline. As the slope of the [Ca]-nullcline changes, it shifts its intersection with the *x*′ = 0 nullcline. The figure highlights how these changes in the nullclines result in different bifurcations as Δ_Ca_ is varied.

In Figure 5B, it can be seen that the application of positive *I*_*app*_ currents shifts the nullcline *x*′ = 0 to the right and bends the SNIC-curve in the same direction, thus increasing the overall excitability of the neuron. On the other hand, negative *I*_*app*_ currents have the opposite effect by decreasing the excitability of the neuron, shifting the nullcline *x*′ = 0 to the left and further bending it. This results in a shrinking of the oscillatory domain and a lower stable hyperpolarized branch.

While the local effect of *I*_*syn*_ on the SNIC-curve is qualitatively similar to the application of the constant current, the overall outcome of the synaptic drive on slow dynamics is profoundly different and significant. One can see from Panel E that increasing the inhibitory current (with *E*_*rev*_ = −70mV) shifts the nullcline *x*′ = 0 to the left, as well as straighten its shape and eliminates its upper knee points. Figure 5F illustrates how the application of an increasing excitatory current translates the location of the SNIC-curve and reshapes the nullcline *x*′ = 0, eliminating hysteresis and hence bursting activity.

There is a simple explanation for the different effects of *I*_*app*_ and *I*_*syn*_. The latter term can be factored into two sub-terms: constant *g*_*syn*_ *E*_*rev*_ which can be treated as some constant current *I*_*app*_, positive or negative, depending in the sign of *E*_*rev*_ and a time-variable term −*g*_*syn*_ *V*(*t*), which also becomes fixed at a voltage steady state, but its value varies along the steady state nullcline *x*′ = 0. As we have seen above that this term always makes the hysteresis shrink by a shear-like transformation of the nullcline, thus overwhelming the opposite effects induced by *I*_*app*_. We stress that the difference between applications of the constant current *I*_*app*_ and synaptic current *I*_*syn*_ has profound implications for the interpretation of electrophysiological experiments.

A careful analysis of Figure 5E reveals the absence of overlap between the unstable (dotted) branch of the *x* nullcline and a given value of the external current *I*_*app*_. This factor alone precludes the possibility of latent bursting, as the presence of hysteresis exists, but is not aligned with the tonic spiking manifold. Nevertheless, it is advisable to incorporate an additional buffer when *x* is not much faster than [*Ca*], as the jump in the trajectory is delayed after passing the catastrophe at the knee point.

**FIGURE 6:**
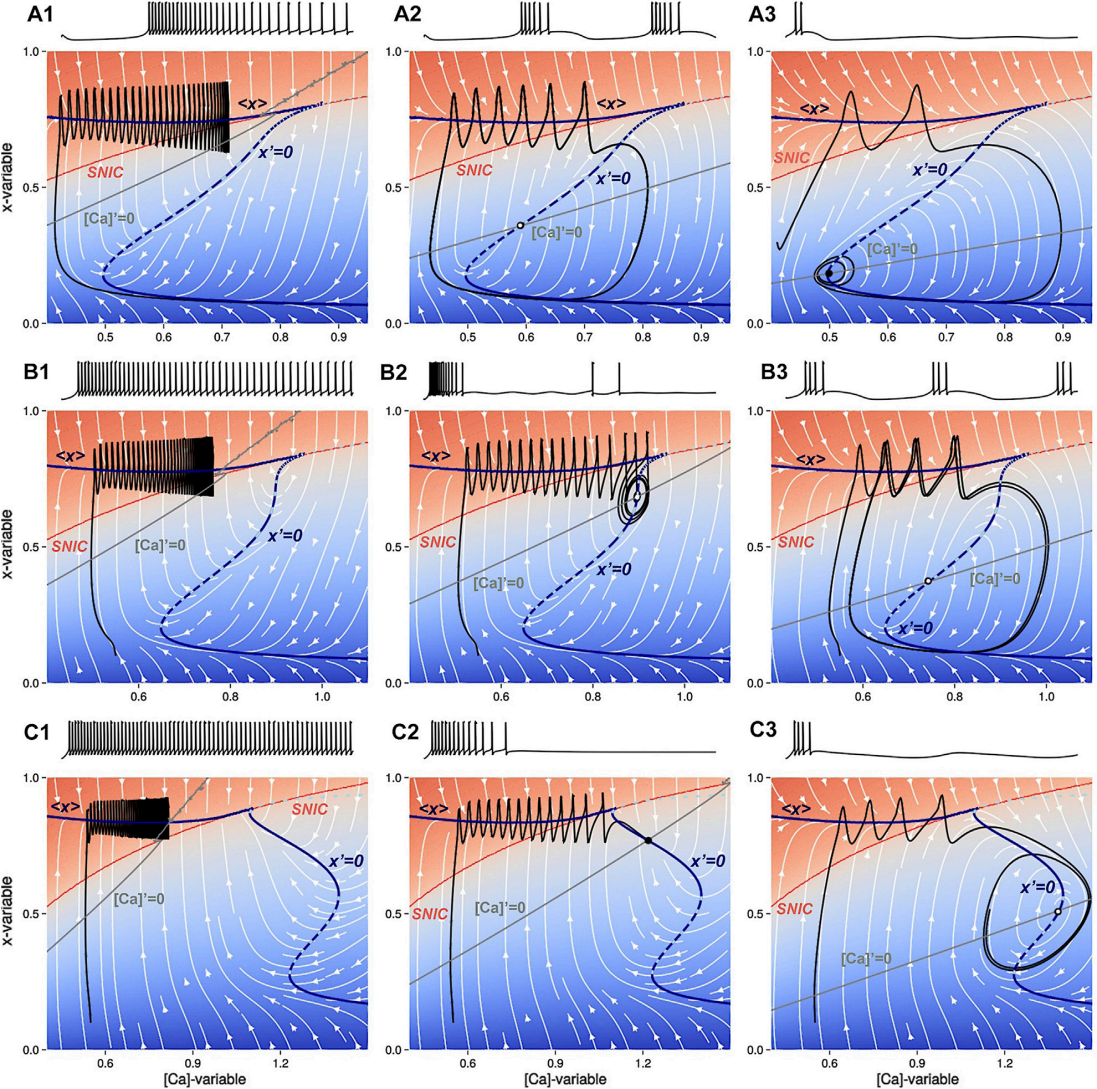
The voltage traces of the slow ([Ca], *x*)-phase plane are shown superimposed with a white stream-plot. There are three different panels, each showing the effects of different values of the parameters Δ_*x*_ and Δ_*C*a_. The top panels **(A1–A3)** show the intermediate bursting regime at Δ_*x*_ =0. Panel A1 shows tonic-spiking activity at Δ_Ca_ =−60mV, with a latent hysteresis due to the overlap of the *x*-nullclines. Panel **(A2)** shows a burster configuration at Δ_Ca_ =0mV. Panel **(A3)** shows a trajectory near the subcritical AH bifurcation with decaying subthreshold oscillations near the lower knee point of the nullcline *x*′=0 at Δ_Ca_ =120mV. The middle panels **(B1–B3)** show transient chaos en route to bursting at Δ_*x*_ =−1.4mV. Panel **(B1)** shows tonic-spiking activity at Δ_Ca_ =−60mV. Panel **(B2)** shows transient chaos for Δ_Ca_ =−32mV before a subcritical AH bifurcation. Panel **(B3)** shows a bursting trajectory at Δ_Ca_ =40mV after the unstable limit cycle disappears. The bottom panels **(C1–C3)** show that there is no occurrence of bursting for Δ_*x*_ =−4 where the SiN-model transitions from tonic-spiking (Panel **C1**) at Δ_Ca_ =−100mV directly to the quiescent state (Panel **C2**) at Δ_Ca_ =0mV and finally to subthreshold oscillations (Panel **C3**) at Δ_Ca_ =105mV. The limit cycle emerges and collapses sequentially through two supercritical AH bifurcations near the high and low knee points, respectively, on the nullcline *x*′=0.

To illustrate the difference in the parameter regimes of the SiN-model (see its bifurcation diagram in Figure 3), we choose Δ_Ca_ as a bifurcation parameter and explore how its variations are correlated with specific bifurcations and how they underlie the transitions between neural activity types. One can see from Figure 6 that the appearance and disappearance of the knee points in the nullcline *x*′ = 0 and the corresponding changes in the slope of the [Ca]-nullcline near the intersection point with the nullcline *x*′ = 0 are directly linked to the bifurcations and the transitions in neural activity patterns. As Δ_Ca_ increases, the knee points of the nullcline *x*′ = 0 may disappear, leading to a change in the stability of the adjacent equilibria, resulting in either the loss of tonic-spiking or the appearance of new periodic orbits, or a combination of both. These bifurcations are responsible for the re-arrangements of the phase-plane, which is why the ([Ca], *x*)-phase plane must be monitored carefully to understand the mechanisms of the transitions between neural activity patterns.

We consider the case where the SiN-model exhibits tonic-spiking activity as shown in Figure 6A1. Note from this panel that after the trajectory crosses above the SNIC-curve, it begins to zigzag (spike) and move towards the stable orbit located near the nullcline [Ca]′ = 0 (represented by the grey line). As Δ_Ca_ increases further, the nullcline [Ca]′ = 0 turns clockwise past the SNIC-curve. To proceed with the analysis, we must consider two cases separately.

• Case 1: the average nullcline ⟨*x*⟩ connects to an unstable branch of the nullcline *x*′ = 0. Then, tonic-spiking activity transforms into bursting activity as occurs in the original Plant model with Δ_*x*_ = 0mV. This case is illustrated in Figure 6A2.
• Case 2: the average nullcline ⟨*x*⟩ connects to a stable branch of the nullcline *x*′ = 0. Then tonic-spiking activity transitions to the quiescent state directly as occurs at the level Δ_*x*_ = −4mV. This case is illustrated in Figure 6B2.

Figures 6A1-A3 show how the ([Ca], *x*)-phase plane changes as Δ_Ca_ is increased at Δ_*x*_ = 0mV, beginning with tonic-spiking activity. The stable periodic orbit in the fast subsystem vanishes through the SNIC bifurcation, resulting in a stable equilibrium state in the slow subsystem. As Δ_Ca_ decreases further, the nullcline [Ca]′ = 0 intersects with the unstable section of the nullcline *x*′ = 0 and the SiN-model starts bursting due to periodic alternations between tonic-spiking and quiescence phases. When Δ_Ca_ decreases even further, the intersection moves below the bottom knee point, resulting in hyperpolarized quiescence. This transition occurs in two stages: first, the bursting limit cycle shrinks and the neuron exhibits small-amplitude subthreshold oscillations, then the limit cycle collapses through a supercritical AH bifurcation near the knee.

Figures 6B1-B3 represent three parallel transition stages as Δ_Ca_ is increased at Δ_*x*_ = −1.4mV, before the Bautin point. The system starts off as a tonic spiker as before, see Figure 6B1. As the intersection between the two slow nullclines reaches the SNIC-curve, the system undergoes a subcritical AH bifurcation creating a stable equilibrium bounded by an unstable limit cycle (Figure 6B2). On the exterior of the limit cycle, there is transient bursting. As Δ_Ca_ increases further, the unstable limit cycle and the basin of attraction of the stable equilibrium grow and shrink, until the unstable manifold of the unstable orbit separates from the chaotic bursting, leading to bistability. As the unstable limit cycle finally disappears in a second a subcritical AH bifurcation, stable bursting emerges Figure 6. Increasing [Ca]′ = 0 further sends the intersection below the bottom knee point leading to quiescence (Figure 6A3).

Figures 6C1-C3 demonstrate the behavior change as Δ_Ca_ increases at Δ_*x*_ = −4mV. As the intersection of the two slow nullclines lowers below the SNIC-curve and crosses the stable section of the nullcline *x*′ = 0, the neuron becomes quiescent (Figure 6C1). As Δ_Ca_ continues to increase, a supercritical AH-bifurcation occurs, marking the start of subthreshold oscillations as the limit cycle does not reach the SNIC-curve in the ([Ca], *x*)-phase plane (Figure 6C2). Finally, when the nullcline [Ca]′ = 0 lowers below the bottom knee point, the subthreshold oscillations are stopped by the hyperpolarized quiescent state, as depicted in Figure 6C3.

The behavior of the system is determined by the shape of the *x*-nullcline in the phase plane. The [Ca]′ = 0 nullcline is controlled by the Δ_Ca_ parameter, and decreasing this parameter causes the nullcline to turn counter-clockwise. If this nullcline intersects the stable section of the *x*′ = 0 nullcline, the intersection becomes a stable equilibrium state of the SiN-model. If the [Ca]′ = 0 nullcline continues to turn counter-clockwise, it will eventually intersect the average ⟨*x*⟩ nullcline, which triggers a bifurcation sequence that can result in the transition from bursting to quiescent to tonic-spiking behavior. This bifurcation sequence can be explained by comparing the phase plane diagram to the bifurcation diagram in Figure 3.

• The bifurcation diagram in Figure 3 suggests that there are three different transition routes through which the swim interneuron model can undergo as Δ_*Ca*_ is increased within [-80, 50]mV range: transition i) from tonic-spiking to bursting activity directly at level Δ_*x*_ = 0, or ii) additionally throughout the quiescent state at Δ_*x*_ = −2, or iii) from tonic-spiking to the quiescence only at Δ_*x*_ = −4.

### 2.5 Neuron bursting in response to synaptic inhibition

**FIGURE 7:**
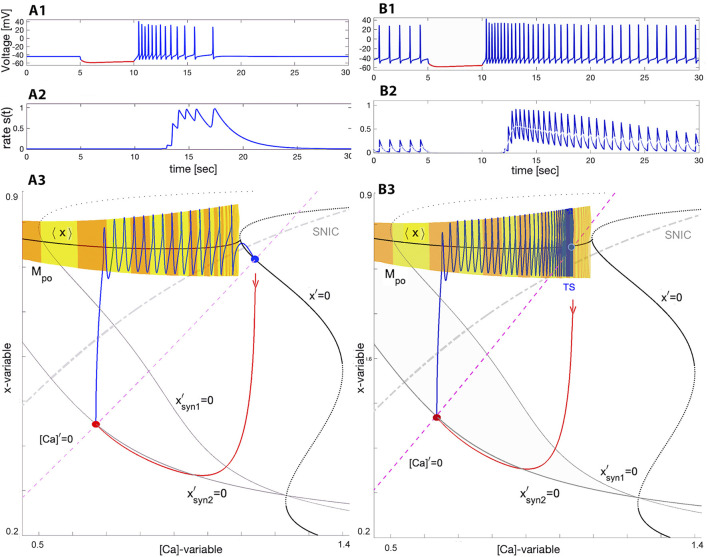
Voltage trace showing quick post-inhibitory rebounds followed by the spike frequency adaptation in the swim interneuron model returning to the stable quiescent state at (Δ_Ca_ =−20mV, Δ_*x*_ =−4mV) in Panel **(A1)** or to tonic-spiking activity at (Δ_Ca_ =−35mV, Δ_*x*_ =−4mV) in Panel B1 after it becomes hyperpolarized by an inhibitory *synaptic* current perturbation −*g*_syn_ (*V*(*t*)+80). Panels **(A2, B2)** show the time evolution of the neurotransmitter release rates matching the spike frequency changes resulting in stronger summation in the logistic synapses (introduced in next section Eq. 22). Panels: **(A3, B3)** show the ([Ca], *x*)-phase projection showing the tonic-spiking manifold *M*_po_, and the unperturbed Σ-shaped nullcline *x*′=0 superimposed with two perturbed ones $x_{\text{syn}1\text{,}2}^{\prime }=0$ straightened by the application of inhibitory *synaptic* current perturbations with *g*_syn_ =0.5 and 1 (left black line). **(A3)** Forced transition from the depolarized quiescent state (blue dot) at the intersection point of the nullcline [Ca]′=0 (pink dashed line) with a stable branch on *x*′=0 towards the stable quiescent state of the neuron in normal conditions, while the red dot on the nullcline $x_{\text{syn}2}^{\prime }$ at *g*_syn_ =1 corresponds to a hyperpolarized steady state emerging temporarily due to the force by the inhibitory synaptic current pulse. Panel **(B3)** depicts the response to the same perturbation of the swim interneuron model transitioning back to the stable periodic orbit at Δ_Ca_ =−35mV, located on the manifold *M*_po_ near the intersection point of the average curve ⟨*x*⟩ and the calcium nullcline [Ca]′=0 above the SNIC-curve in the ([Ca], *x*)-projection.

In order to understand how the network bursting occurs, it is helpful to consider the transients generated as synaptic drive is switched on and off. The reciprocation of these transients provides an explanation for network bursting. Figure 7 illustrates the responses of quiescent (panel A with Δ_Ca_ ≥−20mV) and tonic-spiking (panel B with Δ_Ca_ ≤ −35mV) interneurons after they are temporarily hyperpolarized by a 5 [sec]-long inhibitory synaptic current (episodes in red). The voltage traces in Figure 7A1, B1 show that after the perturbation the SiN-model exhibits a *fast* post-inhibitory rebound with a high spike frequency initially, followed by a *slow* adaptation while returning to their respective attractors. Note that the slow spike frequency adaptation observed in the voltage traces is driven by the slow [Ca]-dynamics in the model.

Consider the swim interneuron model under perturbation caused by a pulse of the inhibitory synaptic current *I*_*syn*_ = *g*_syn_ (*V*(*t*) + 80). The pulse duration of 5 s is long enough for the SiN-model to converge onto a newly perturbed stable state, as can be seen from the voltage traces. In addition to the original, unperturbed nullcline *x*′ = 0, Figure 7A3, B3 depict two additional perturbed nullclines, labelled $x_{\text{syn}1}^{\prime }=0$ and $x_{\text{syn}2}^{\prime }=0$ , corresponding to two different *g*_syn_-values, 0.5 and one respectively. The intersection point (red dot) of the nullcline [Ca]′ = 0 with the stable nullcline $x_{\text{syn}2}^{\prime }=0$ at *g*_syn_ = 1 is a stable equilibrium state of the inhibited neuron. Shown in red is a phase trajectory forced to quickly transition from the unperturbed stable equilibrium state (blue dot) in Figure 7A3, or from a periodic orbit in Figure 7B3 towards the inhibited or perturbed steady state (red dot). This steady state persists as long as the inhibitory current lasts.

As soon as the inhibition is removed, the SiN-model responds with a fast PIR associated with the trajectory (blue line) in the ([Ca], *x*)-phase plane that takes off nearly vertically from the perturbed steady state towards the tonic-spiking manifold *M*_po_. Having landed onto *M*_po_, it slowly transitions back to its original state along the manifold with a gradually decreasing spike rate. Note that the decreasing size of the steps between spikes on the manifold *M*_po_ in the phase plane is deceptive, because orbits to the left of the *M*_po_ populate the phase plane less densely, even though they are slower. This is simply a consequence of exponential convergence to the attractor in the slow subsystem and is not indicative of the speed of the periodic orbit in the fast subsystem.

To summarize the discussion on the cellular properties:

• Quiescent and tonic-spiking neurons in isolation can produce an episodic burst after recovery from forced inhibition.
• The stronger the inhibition is, the greater spike frequency becomes in the post-inhibitory rebound due to smaller [Ca]-values associated with the forced state in the neuron, and the more pronounced the slow spike frequency adaptation is in the corresponding voltage trace.
• The duration of the post-inhibitory rebound and the adaptation speed are determined by the change rate [Ca]′ of the calcium concentration, as well as the distance to travel back to the initial state of the neuron, which is determined by the position of the nullcline [Ca]′ = 0 (due to Δ_Ca_) in the ([Ca], *x*)-phase plane.

In the following sections, we will show how the cellular dynamics enhanced with a strong PIR and the slow spike frequency adaptation can coordinate with slow synaptic dynamics to generate emergent network-level bursting in two generic types of building blocks for rhythm-generating circuits.

### 2.6 Modeling synapses

The synapses in *Melibe* and *Dendronotus* may be either fast or slow. Slow synapses are of particular interest, as they occur on the same timescale as network oscillations and have been reported in the swim CPGs of the sea slugs (Watson et al., 2002; Watson and Newcomb, 2002; Thompson and Watson, 2005; Sakurai et al., 2011). In order to be able to accurately control the frequency response of the synaptic gating variable we introduce a new *logistic* model that can describe both delayed and slow synaptic summation, which can act as an efficient high-pass filter. We briefly compare the logistic synapse’s properties with similar synapses from literature, namely, the *α*-synapse, (Rall, 1967; Wang and Rinzel, 1992), higher order kinetics (Destexhe et al., 1994a; Golomb et al., 1996; Destexhe et al., 1998), and a dynamic synapse (Hill et al., 2001).

### 2.7 Fast threshold modulation

(FTM). This simple paradigm (Wang and Rinzel, 1992; Kopell and Somers, 1993) adequately describes fast synapses. In a FTM framework, the synaptic activity is turned “on” or “off” instantaneously. This directly utilizes the aforementioned *f*_*∞*_ function with the presynaptic membrane potential *V*_*pre*_:

$$S\left(t\right)=f_{\infty }\left(V_{\mathrm{pre}}\left(t\right)\right).$$ (13)

Such fast synapses happened to be useful for understanding synchronized neuronal activity, including bursting with inhibitory synapses, and multistability in small, weekly coupled neural networks (Belykh and Shilnikov, 2008; Shilnikov et al., 2008; Jalil et al., 2010; Wojcik et al., 2011; Wojcik et al., 2014; Schwabedal et al., 2016; Collens et al., 2020; Kelley and Shilnikov, 2020; Pusuluri et al., 2020). Moreover, this assumption has the benefit of simplifying the network dynamics, facilitating both analysis and simulation (Jalil et al., 2013; Schwabedal et al., 2014; Lodi et al., 2019).

As FTM synapses do not have any temporal dynamics, the FTM paradigm does not suit the kinetics of slow synapses. When temporal dynamics of synapses become pivotal in neural circuitry, the lack of dynamics in the FTM becomes a critical limitation for modeling. This is the case where the synaptic strength changes dynamically with time and can sum up at higher ranges of spike frequency in a pre-synaptic neuron to cause substantially nonlinear effect, inhibitory or excitatory, on a post-synaptic neuron. It was established in Refs (Beveridge, 2009; Sakurai and Katz, 2016). that both swim CPGs in the given sea slugs rely on synaptic summation to maintain slow bursting oscillations. So, whenever the synaptic activation changes gradually, the time-varying dynamics governing the synaptic current in Eq. 20 is to be described by an ODE or even an ODE system to account for other nonlinear synaptic qualities like potentiation or fatigue, for example.

**FIGURE 8:**
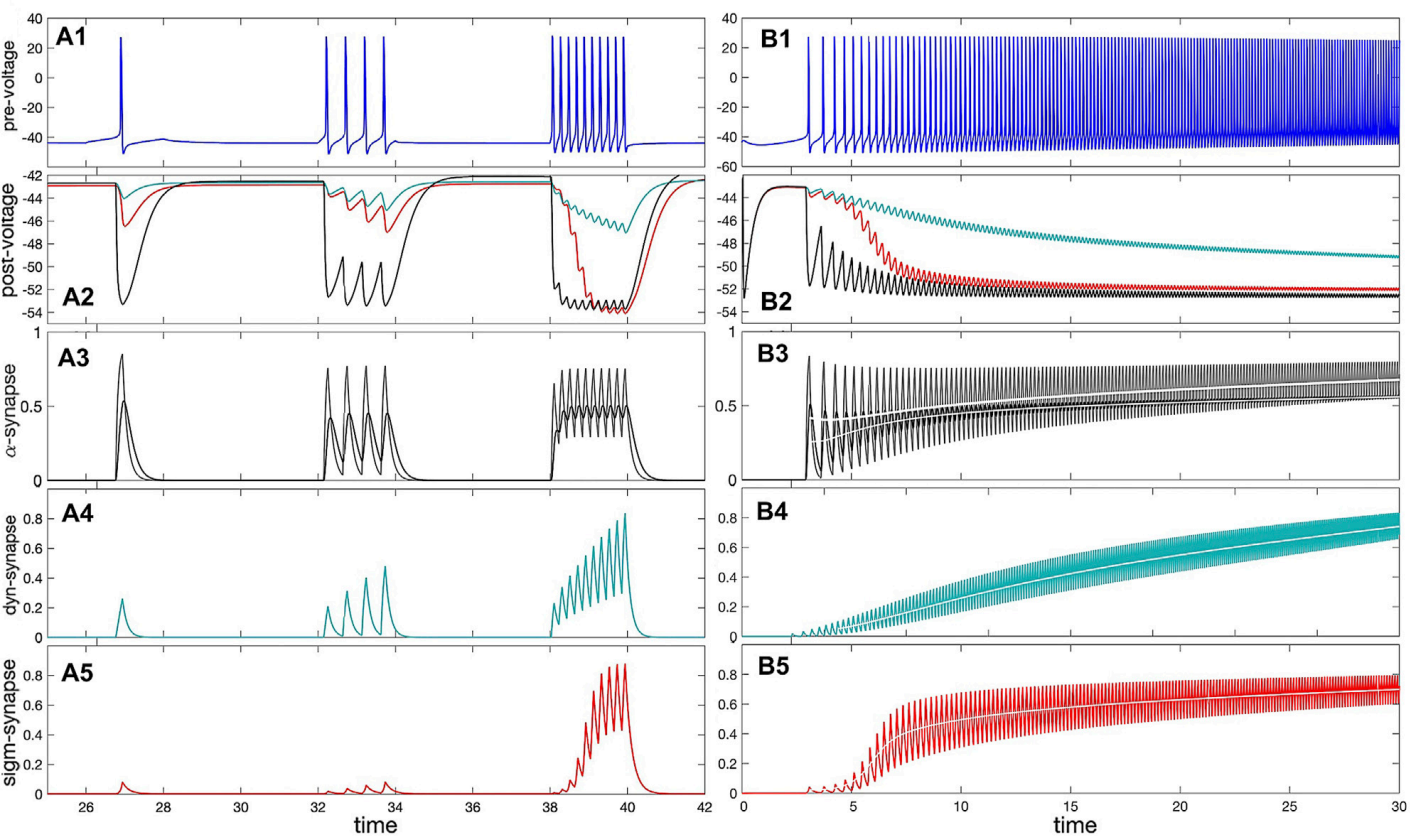
Panel **(A1)**: voltage trace of the pre-synaptic (quiescent) interneuron model receiving depolarizing (positive) square pulses of increasing amplitude. **(A2)** IPSP on the voltage trace of the post-synaptic (quiescent) interneuron model receiving inhibitory (hyper-polarizing) synaptic currents from the pre-synaptic interneuron in **(A1)** modeled using the *α*-synapse of the first-order kinetics **(A3)**, the slow dynamic **(A4)** and logistic **(A5)** synapses. **(A3)** The probability/rate of the neurotransmitter release in the *α*-synapse using the first order (black line) with *α* =0.01 and *β* =0.008, and second order (cyan line) kinetics in response to a single spike and spike trains in the trace shown in Panel **(A1)**. Panels **(A4, A5)** demonstrate stronger responses or accumulation in the dynamic (*τ*_*M*_ =1200) and logistic synapses. **(B1)** Voltage trace shows a gradual adaptation/transition (due to slow [Ca]-dynamics) of the pre-synaptic interneuron transitioning from an initially quiescent state to its native tonic-spiking activity with a high frequency at Δ_Ca_ =−70mV. **(B2)** Voltage trace revealing responses of the quiescent post-synaptic interneuron receiving synaptic inhibitory current from the pre-synaptic interneuron (Panel **B1**) modeled using the *α*-synapse **(B3)**, the dynamic **(B4)** and logistic **(B5)**. Panels **(B3–B5)**: increasing rate of the neurotransmitter release in the *α*-, dynamical and logistic synapses correlating with the higher spike frequency in the pre-synaptic interneuron in Panel **(B1)**. The white lines show the corresponding average.

**FIGURE 9:**
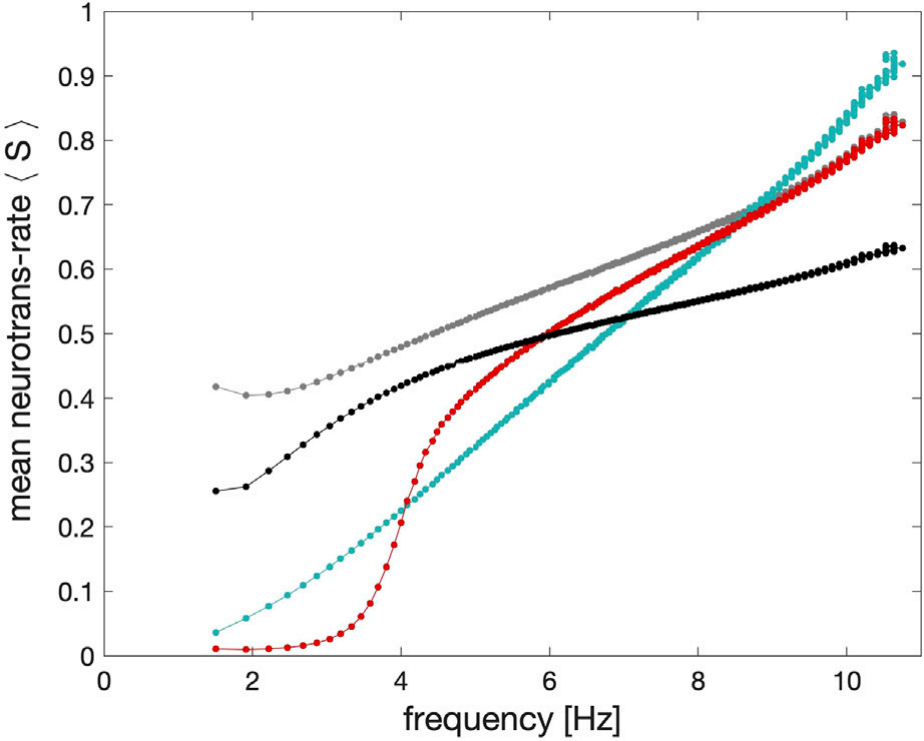
Shape comparison of the *average* synaptic probabilities or neurotransmitter release rates ⟨*S*⟩ in the models of the *α*-synapse with the first (grey dots) and second order (black dots) kinetics, the dynamic (cyan dots) and the logistic synapses (red dots) plotted against the spike frequency in the pre-synaptic neuron. The key feature of the logistic synapse is its inflection point.

The relationship between pre-synaptic frequency and neurotransmitter release is computed for each synapse from trajectories of a pre-synaptic cell coupled to a post-synaptic cell. The frequency of the pre-synaptic cell is increased over time by slowly decreasing the Δ_Ca_ parameter. For each spike over this trajectory, the frequency is calculated as the reciprocal of the inter-spike interval, and the average neurotransmitter rate, *∠S*⟩ is computed from the time-average of the synaptic gating variable *S* over the same spike. The time series demonstrating this comparison is pictured in Figure 8, and the curves showing the relationship between pre-synaptic frequency and <S> are shown in Figure 9.

### 2.8 α-synapse

Adopting the phenomenological approach taken by Hodgkin and Huxley, Wang and Rinzel (Wang and Rinzel, 1992) proposed what is now commonly referred to as an *α*-synapse. Its activation is meant to mimic the profile of an *α*-function given by *t* ^*p*^ *e* ^−*t*^, where *p* is a positive integer. The idea of such *α*-synapses is rooted in the pioneering computational work by W. Rall (Rall, 1967), who studied and modeled various aspects of synaptic potentials.

The dynamic equation describing the rate of change of the *S*(*t*)-variable in the *α*-synapse is given by

$$S^{\prime }\left(t\right)=\alpha \left(1-S\right)f_{\infty }\left(V_{\mathrm{pre}}\right)-\beta S$$ (14)

with some positive *α* and *β* constants. So, when *V*(*t*) is below the synaptic threshold Θ_*syn*_ and hence *f*_*∞*_ = 0, then the synaptic probability *S*(*t*) exponentially decreases to zero as *e* ^−*βt*^. During an action potential as long as *V*(*t*) ≥Θ_*syn*_, then *S*(*t*) is approaching the equilibrium state (*α*/(*α* + *β*)) exponentially fast as *e* ^−(*α*+*β*)*t*^. Note that Eq. 14 is sometimes referred to as an *α* − *β*-synapse with the first-order kinetics (Destexhe et al., 1994b).

If *α* and *β* values are on the same order of magnitude, say, 0.1, then the *α*-synapse is as fast as an FTM (Jalil et al., 2012; Alaçam and Shilnikov, 2015; Baruzzi et al., 2020; Baruzzi et al., 2021). Decreasing *β* by one order of magnitude ∼0.01 makes the synapse sufficiently slow and stronger as it can accumulate and demonstrate the pronounced summation with monotonically increasing *S*(*t*) on average, see Figure 8A3, B3 (grey lines), in response to trains of fast spikes in the pre-synaptic neuron. Typical values for the *α*-synapses in the swim CPGs are *α* = 0.01, while *β* is set in the range $\left[0.001,\;0.0005\right]$ for slow synapses, and $\left[0.01,\;0.1\right]$ for fast ones. Decreasing proportionally *α* and *β* makes the time progression of *S*(*t*) smoother with smaller amplitude variations.

The dynamics of the *α*-synapse can be further enhanced by adding higher order synaptic kinetics that is modeled by an ODE system with the feed-forward structure of equations like Eq. 14 (Destexhe et al., 1994a; Golomb et al., 1996; Destexhe et al., 1998):

$$S_{1}^{\prime }\left(t\right)=\alpha_{1}\left(1-S_{1}\right)f_{\infty }\left(V_{\mathrm{pre}}\right)-\beta_{1}S_{1},$$ (15)

$$S_{2}^{\prime }\left(t\right)=\alpha_{2}\left(1-S_{2}\right)\;S_{1}\;\;\;\;-\;\;\;\;\;\;\;\beta_{2}S_{2},$$ (16)

$$S_{3}^{\prime }\left(t\right)=\alpha_{3}\left(1-S_{3}\right)\;S_{2}\;\;\;\;-\;\;\;\;\;\;\;\beta_{3}S_{3},\;\text{and so on,}$$ (17)

with the same or different time constants *α*_*i*_ and *β*_*i*_ in each equation. In the case of same time constants, the synapse model of higher kinetics creates a smoothing effect and dampens the effect of individual spikes within a burst in the pre-synaptic neuron, see Figure 8A3, B3 (black lines).

### 2.9 Dynamic synapse

Regarding synaptic plasticity, the accumulation of the slow *α* synapse is a poor model because it is not compatible with pronounced postsynaptic potentials. One approach to creating facilitated synapses is to introduce a modulating variable *M* which operates on a different timescale from *S*. This approach was originally introduced to model a spike-mediated synaptic current (Hill et al., 2001)

$$\;I_{\mathrm{syn}}=g_{\mathrm{syn}}\;S\left(t\right)\;M\left(t\right)\;\left(V_{\mathrm{post}}\left(t\right)-E_{\mathrm{rev}}\right),$$ (18)

where *S*(*t*) can be borrowed from the fast *α*-synapse (Eq.14), or from the FTM-synapse (13), while the rate of change of the *M*(*t*)-variable is supposed to be quite slow

$$M^{\prime }\left(t\right)=\frac{f_{\infty }\left(V_{\mathrm{pre}}\right)-M}{\tau_{M}}$$ (19)

with a large time constant *τ*_*M*_ ∼ 10^3^ or greater. We will discuss the temporal characteristics of the dynamic synapse below.

We also briefly mention an additional modeling technique to alter the temporal characteristics of synaptic current while leaving dynamics of the synaptic probability *S*(*t*) intact. The idea, which was also borrowed from the HH-formalism and originally intended for calibration of conductance values, is to use higher powers of *S* in the synaptic current equation *g*_*syn*_ *S* ^*p*^(*V* − *E*_*rev*_), *p* = 2, 3 …. The objective is to reshape the ascending concave-down course of *S*(*t*) at its initial phase to a concave-up one with a following inflection point in the time-progression due to the *S* ^*p*^(*t*)-term (0 ≤ *S*(*t*) ≤ 1). This modification is supposed to cause initial delays and result in a less rapid/steep build-up in such a slow synapse.

The synaptic current *I*_*syn*_ in the post-synaptic neuron is modeled as follows:

$$\;I_{\mathrm{syn}}=g_{\mathrm{syn}}\;S\left(t\right)\;\left(V_{\mathrm{post}}\left(t\right)-E_{\mathrm{rev}}\right),$$ (20)

where *g*_*syn*_ is a maximal conductance, *S*(*t*) is a synaptic gating variable, *V*_*post*_ is the membrane voltage in the post-synaptic neurons, and *E*_*rev*_ is a synaptic reversal potential, which can be set at +40mV or −80mV for excitatory and inhibitory synapses, respectively. The sigmoid function *f*_*∞*_(*V*) is defined as

$$f_{\infty }\left(V\right)=\frac{1}{1+e^{-k\left(V_{\mathrm{pre}}-\Theta_{\mathrm{syn}}\right)}},$$ (21)

where the constant *k* determines its derivative at *f*_*∞*_(*V*) = 0.5, the inflection point where *V* = Θ_*syn*_. Here Θ_*syn*_ is treated as the synaptic threshold typically set around +20mV in the middle of spikes, between the spike threshold around −40mV (sodium channels opens) and the spike peak +40mV. Values for *k* are typically set somewhere in a range $\left[0.5,\;50\right]$ , and as the value of *k* is set large, the function becomes a continuous approximation of the Heaviside step function, where the function *f*_*∞*_ remains close to zero as long as *V*_*pre*_(*t*) < Θ_*syn*_, and quickly jumps to 1 after the voltage in the presynaptic neurons exceed the synaptic threshold.

Most slow synapses act through complex sequences of nonlinear interactions including secondary messenger cascades, which can delay the neurotransmitter release in pre-synaptic neurons or their binding with neurotransmitter receptors in post-synaptic ones. In such cases it is sometimes desirable to introduce a dynamic delay or some low spike frequency filter without explicitly modeling every stage of postsynaptic intracellular signal transduction in terms of state variables.

### 2.10 Logistic synapse

For this purpose, we introduce a new synapse model, which we call the logistic synapse, named after its similarity to the logistic model of population growth, where the probability *S*(*t*) is governed by a single ODE:

$$S^{\prime }\left(t\right)=\alpha \;S\;\left(1-S\right)\;f_{\infty }\left(V_{\mathrm{pre}}\right)-\beta \left(S-S_{0}\right),$$ (22)

where a constant 0 ≤ *S*_0_ ≤ 10^−3^ can be viewed as some spontaneous neurotransmitter release rate on average in the pre-synaptic neuron. The key feature of this modeling approach is the term *S* (1 − *S*) which provides: (i) means to control a latency of the synapse in the initial ascending phase of summation, and hence (ii) a logistic or sigmoid-like shape of the *S*(*t*)-variable determining the proportion of open channels. With this logistic model, we obtain a desired nonlinear dependence of the strength of the synaptic current on the spike frequency in the pre-synaptic neurons to match the experimental studies on the swim CPGs. In addition, with the logistic synapse acting as a high-pass filter we can further explore the role of such time-varying synaptic coupling on rhythmogenesis and its robustness in 2-cell building blocks of two types considered below.

In Figure 9 we show the frequency response of the logistic synapse compared with alternative approaches to synaptic modeling to determine the dependence of the strength of the synapse models on the spike frequency in the pre-synaptic neurons in the long run, skipping initial transients. One can see from this diagram that in the case of the *α*-synapse of the first (grey) and second (black) order kinetics there is a threshold around 2.5–3Hz, after which its strength escalates nearly instantaneously to increase slowly with the higher spike frequency. Observe that the dependence of the strength of the dynamic synapses on the spike frequency is nearly monotonically linear while the logistic synapse remains weak as long as the spike frequency in the neurons stays below 3 Hz. With the higher spike frequency, its efficiency and strength rapidly grow within the spike range 3–6 Hz.

**FIGURE 10:**
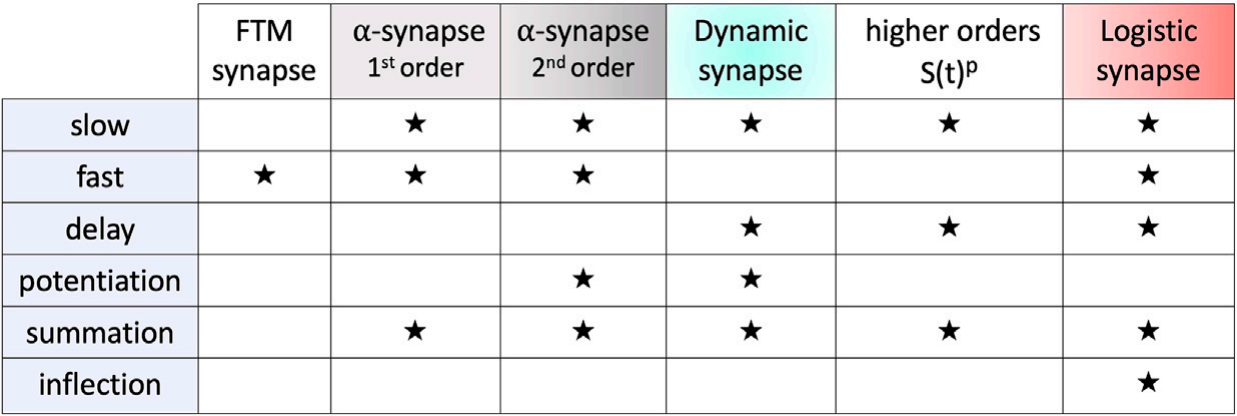
Summarization of the key properties such as delay, potentiation, summation and inflection, of the fast and slow synapse models.

In Figure 10, the term *inflection* refers to the concavity change point in the sigmoid characteristics of the logistic synapse. Here, summation refers to a nearly monotone, solid buildup of I/EPSPs in a voltage trace of the post-synaptic neuron caused and aligned with the spike train in the pre-synaptic one. In contrast, in the case of potentiation the size of I/EPSPs increases with each sequential spike in the pre-synaptic neurons, followed by a reset to a base-line between sequential spikes. The potentiation typically occurs in a synapse model described by an ODE system with diverse fast and slow time-scales, such as Eqs 15–18 or Eqs 18, 19 due to distinct *α*-values on different magnitude orders, say *α*_1_ = 1 and *α*_2_ = 0.1, or *α*_1_ = 1 and a large *τ*_*m*_-constant, respectively. Note that with a higher spike frequency, potentiation may likely morph into summation.

## 3 Dynamics and stability of network bursting in coupled pairs

Below we present two different mechanisms of rhythm generation in such pair-wise networks. The first example is emergent bursting in a reciprocally inhibitory network, reminiscent of Brown’s original HCO, see Figure 1 The second neural pair consists of excitatory and inhibitory neurons (Figure 1B) where the tonic-spiking neuron provides an excitatory drive to the quiescent or less active neuron that subsequently provides a slowly building inhibition. For each network we show several different configurations corresponding to the endogenous behavior of the cells. We discuss the stability and multistability at different parameter values and briefly summarize the mechanism in terms of the coordination of nullclines as they are recurrently driven by synaptic current as described in the methods and models section above.

### 3.1 Half-center oscillator

HCO configurations generate symmetric oscillations that have a phase lag of one-half period. In each of the following HCO figures, the phase plots on the left show the trajectories of two cells, and two nullclines, unperturbed *x*′ = 0 and perturbed or inhibited $x_{\text{inh}}^{\prime }=0$ . These nullclines in each phase plot correspond to the unperturbed and maximal levels of the synaptic drive, respectively in the pre- and post-synaptic neurons alternating sequentially due to the symmetry of the network. The position of the nullcline for a cell at a given time is an interpolation between these two. The movement of the nullclines through the burst cycle can be visualized concretely in Appendix III in Supplementary Material below.

### 3.2 Two quiescent interneurons

**FIGURE 11:**
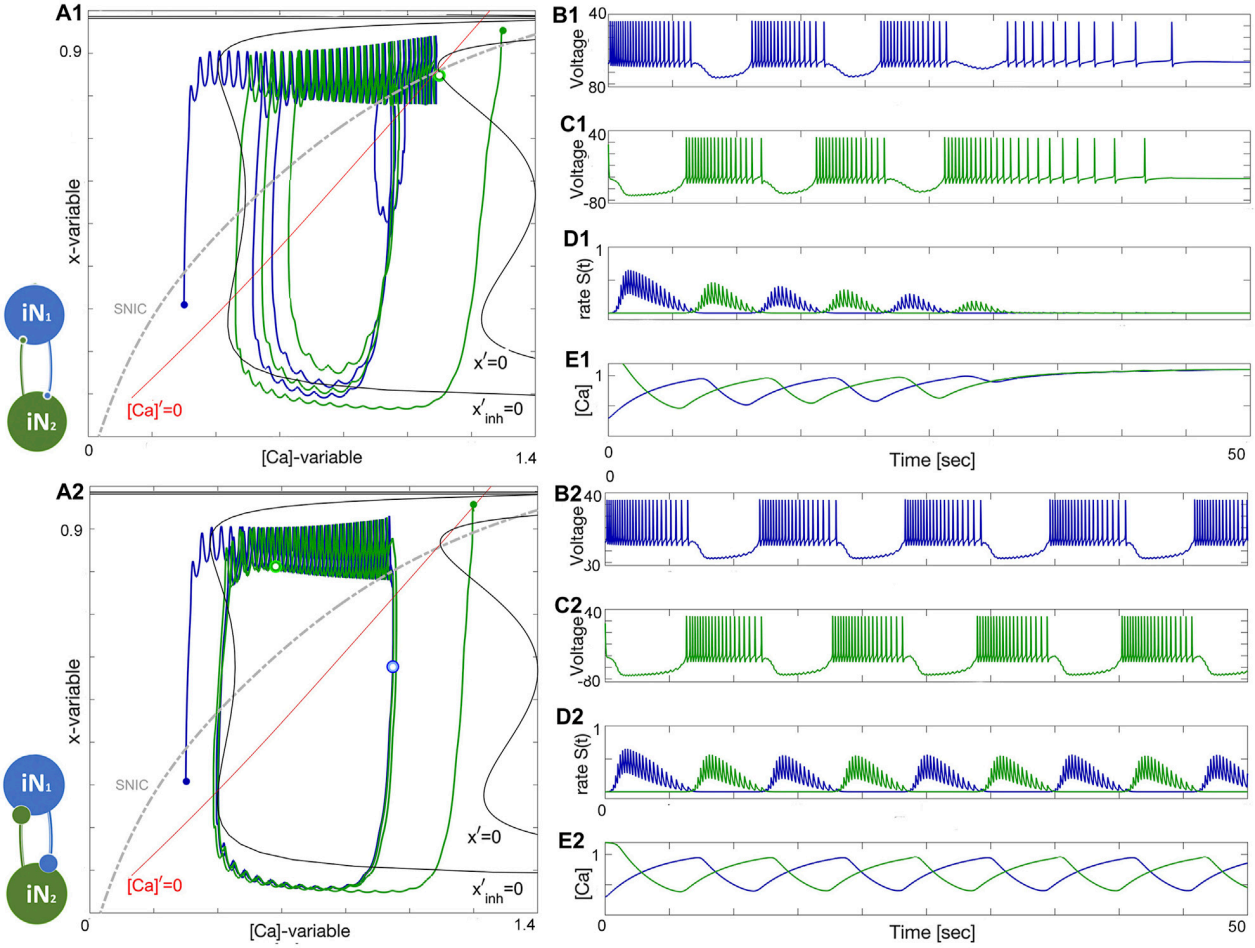
An HCO network constituted of two quiescent interneurons, iN_1_ (blue) and iN_2_ (green) at Δ_Ca_ =−30mV, coupled reciprocally by the inhibitory (denoted by •) logistic synapses. Schematic diagram (left). Panels **(A1–E1)**: insufficient inhibition (*S*(*t*)-traces in Panel **D1**) and/or improper phase-lag (small green and blue dots) between initial phases of the HCO interneurons does not let emerging network-bursting keep the initial momentum and oscillations seize and converge to a quiescent state (voltage traces in Panels **B1, C1**), which is located at the crossing of the nullclines *x*′=0 and [Ca]′=0 (red and black lines, resp.) below the SNIC-curve (as a dotted grey line) in the ([Ca], *x*)-phase plane in Panel **(A1)**. Panels **(A2–E2)**: increasing the reciprocal inhibition strength leads to the onset of self-sustained emergent bursting in the HCO. HCO bursting is associated with a stable cycle in the ([Ca], *x*)-phase plane shown in Panel **(A2)**, which occurs due to the emergent network hysteresis in the driven neuron during the slow hyperpolarized phases near the forced nullcline $x_{\text{inh}}^{\prime }=0$ . This HCO is *bistable*: bringing initial states of the neurons close together will lead to decaying oscillations. The synaptic parameters are *g*_*syn*_ =0.027, *α* =0.05, *β* =0.0051 in the upper panels and *g*_*syn*_ =0.047nS, *α* =0.05, *β* =0.0051 *s* ^−1^ in the bottom panels.

Depending on the coupling strength, a stable oscillatory regime appears. When the inhibition is weak, as in Figure 11, or the initial phases of the constituent neurons are too close, the bursting pattern decays and converges to the steady state at Δ_Ca_ = −30mV. When the strength of inhibition is high, the network exhibits stable oscillations. This is a bistable pattern since both cells are quiescent. Setting the initial conditions for the synaptic variables to zero would result in a silent network regardless of which synaptic parameters are chosen. When the coupling strengths are insufficient to maintain oscillations, but the initial conditions are appropriately chosen, the network bursting persists for several bursts before the transient fades. Since both cells are quiescent, the burst transition may occur through the release mechanism, but since there is never a stable equilibrium, it may also be an escape mechanism. This phenomenon occurs when inhibition is sufficiently strong such that the intersection between the slow nullclines falls on the spiking side of the SNIC curve. On the other hand, the mechanism may be characterized as a post inhibitory rebound since the cell will emit a transient spike train following inhibition.

### 3.3 Two tonic interneurons

The case when both neurons are tonic is similar to the quiescent case, both in mechanism and appearance. The network is illustrated in Figure 11. Both the initial phase and strength must be appropriate as in the previous case. Setting the phases near the borderline of the basin of attraction for network oscillations shows that the oscillations slowly grow in strength and converge to the final attractor. In this case, the escape mechanism cannot play a role since the calcium transient converges to a spiking periodic orbit. The gradual convergence to the burst pattern in 11A2 demonstrates the breadth of the basin of attraction for the bursting rhythm.

**FIGURE 12:**
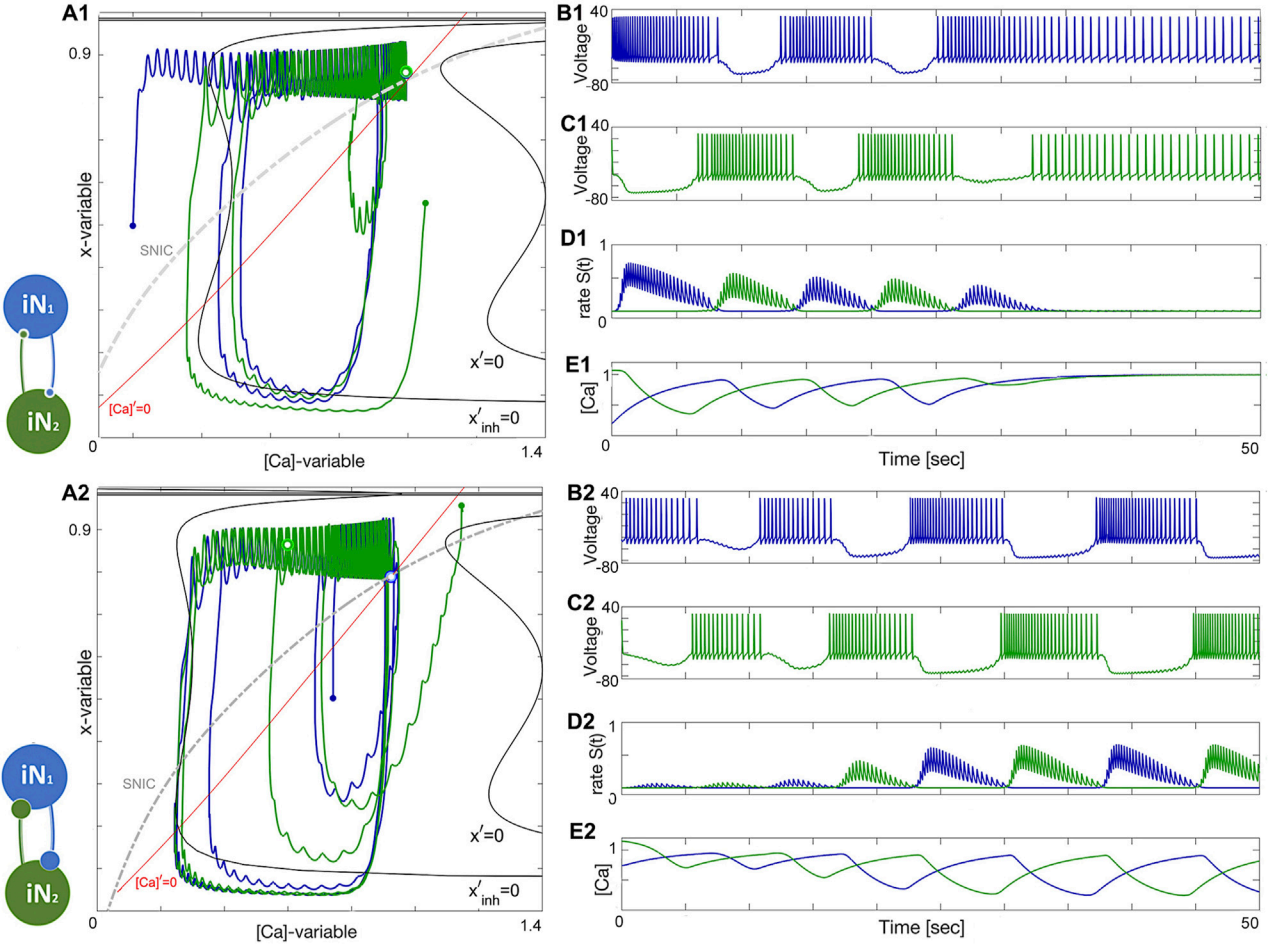
An HCO network constituted of two tonic-spiking neurons at Δ_Ca_ =−40mV. Panels **(A1–E1)**: insufficient reciprocal inhibition and/or phase-lag between initial phases cause network bursting to weaken and make both neurons return to tonic-spiking activity, which can be well-observed in [Ca]-traces (Panel **E1**) converging to some fixed value corresponding to two round periodic orbits indicated by a double dot above the SNIC-curve near the (red) nullcline [Ca]′=0 in the phase plane in Panel **(A1)**. Panels **(A2–E2)**: changing initial phases and/or increasing the reciprocal inhibition leads to the onset of network bursting, corresponding to a stable limit cycle in the ([Ca], *x*)-phase plane shown in panel **(A2)**. This HCO is *bistable*: decreasing inhibition or/and initial phase-lag will lead to similar damping oscillations as in the previous case. The synaptic parameters are *g*_*syn*_ =0.057nS, *α* =0.03, *β* =0.003 *s* ^−1^ in the upper panels and *g*_*syn*_ =0.067nS, *α* =0.03, *β* =0.003 *s* ^−1^ in the lower panels.

Figure 12 shows how network anti-phase bursting evolves asymptotically through a canard-type periodic orbit with small initial amplitude via an AH bifurcation. If the *x*-dynamics are slowed down to match the time scale of the [Ca]-dynamics, stable network oscillations can develop gradually. A1-E1 show that below a bifurcation threshold, network oscillations decline and converge to the tonic-spiking (round) periodic orbit (seen in the ([Ca], *x*)-phase plane in Figure 12A1). The stability of this HCO network bursting is evident in the growth of *S*(*t*) oscillations (Figure 12D2) and calcium concentration (Figure 12E2). However, bistability may result in the absence of oscillations, as shown in Figure 12B1. The network can exist in two states: both neurons exhibit tonic-spiking activity or the network bursts. To initiate network bursting, one option is to have one neuron initially inactive and the other active, or to excite both neurons with an external current or synaptic pulse. This triggers an inhibitory race between them, leading to anti-phase bursting.

### 3.4 “Winner takes all.”

**FIGURE 13:**
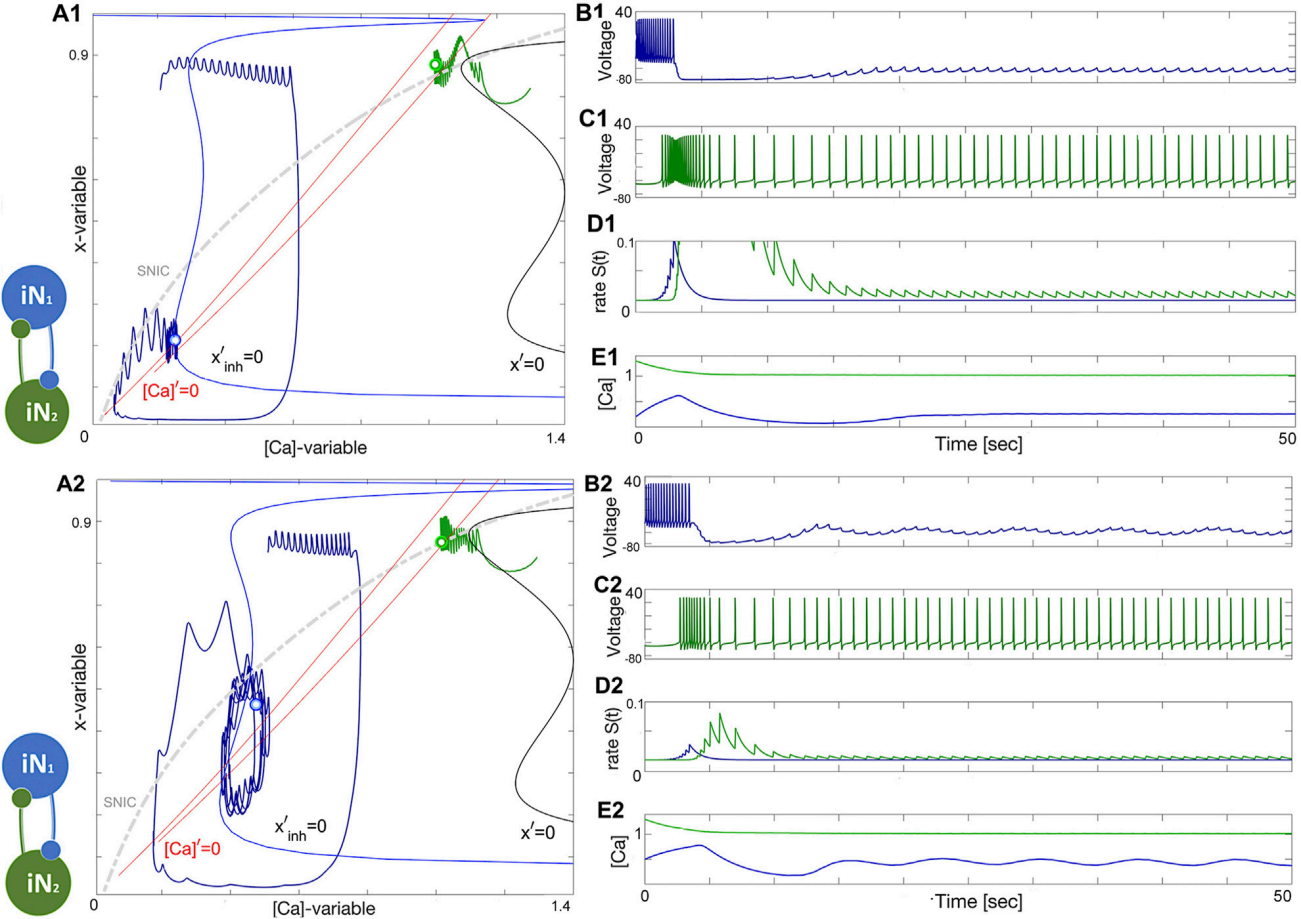
Illustration of the “winner takes all” concept in the HCO network. Panels **(A1–E1)**: The initial positions place the green neuron at Δ_Ca_ =−40mV atop so that its strong and lasting inhibition forces the blue counterpart neuron at Δ_Ca_ =−50mV to stay at a hyperpolarized state near the intersection of the corresponding nullclines [Ca]′=0 and *x*′=0 (next to its bottom knee-point) and under the SNIC-curve in the phase plane as shown in panel **(A1)**. Panels **(A2–E2)**: Weakening inhibition makes the blue neuron becomes less hyperpolarized to produce forced sub-threshold oscillations seen in the voltage traces and corresponding to a limit cycle in the ([Ca], *x*)-phase plane between the two knee-points of the Σ-shaped nullcline *x*′=0 as depicted in Panel **(A2)**. The synaptic parameters are *g*_12_=0.1nS, *α*_12_=0.021, *β*_12_=0.0013*s* ^−1^, and *g*_21_=0.8nS, *α*_21_=0.03, *β*_21_=0.0011*s* ^−1^ in the upper panels and *g*_21_ is reduced to 0.1nS in the lower panels.

To summarize the discussion on HCO-dynamics, in a “winner takes all” scenario, it is common to see variation in the spike frequency of neurons. In this setup, the blue neuron has a high spike frequency at Δ_Ca_ = −50mV while the green neuron has a lower spike frequency at Δ_Ca_ = −40mV. However, as seen in Figure 13A1, the green neuron becomes the winner, inhibiting and shutting down the blue neuron at the forced hyperpolarized state. This occurs when the lasting inhibition is too strong, either due to a larger *α*-value or a lower *β*-value in the logistic synapse. Weakening the green neuron’s inhibition allows the blue neuron to rise, producing sub-threshold oscillations in its voltage trace. These oscillations are associated with a stable network limit cycle in the phase plane, and with further weakening of the green neuron’s inhibition, the amplitude of the sub-threshold oscillations will increase, leading to full bursting episodes.

Le us conclude the HCO section with the following observations:

• HCO bursting with $\frac{1}{2}$ phase-lag is a emergent phenomenon based on the network hysteresis, and originates from transient dynamics in the constituent neurons;
• HCO bursting becomes self-sustained provided that the balance of phases and the balance of amplitudes (coupling) are fulfilled;
• If either balance condition fails, then the HCO bursting falls apart and its constituent neurons come back to their natural states;
• The HCO is a bistable network;
• coupling with logistic synapses makes the HCO-dynamics flexible and fluctuating, and less stiff compared to *α*-synapses;
• HCO can be composed of both (non-identical) quiescent, or both tonic-spiking neurons, or their combinations, which warrants its wide structural stability range, and makes it less dependent on variations of cellular parameters.

### 3.5 Excitatory–inhibitory (E/I) module

The excitatory-inhibitory (E/I) module is analogous to the classic predator-prey relationship and a common block of other similar networks (Venkadesh et al., 2024). The excitatory (blue) neuron acts as the “prey” by providing enough drive for the inhibitory (green) neuron, acting as the “predator,” to generate tonic-spiking activity and slow down the excitation through inhibitory feedback. This results in the excitatory neuron transitioning to an inactive, hyperpolarized phase, causing the predator to lose the drive from the excitatory neuron. The excitatory neuron then returns to its initial state, freeing the prey from inhibition. A weak electrical synapse, or gap junction, is included between the neurons in this E/I module, which was reported in the biological E/I module, a key building block in the swim CPG of the sea slug, *Dendronotus iris* (Sakurai and Katz, 2022), see Figure 1B. Although this gap junction was present, it was not observed to play a significant role in rhythmogenesis, and the network operates well without it.

**FIGURE 14:**
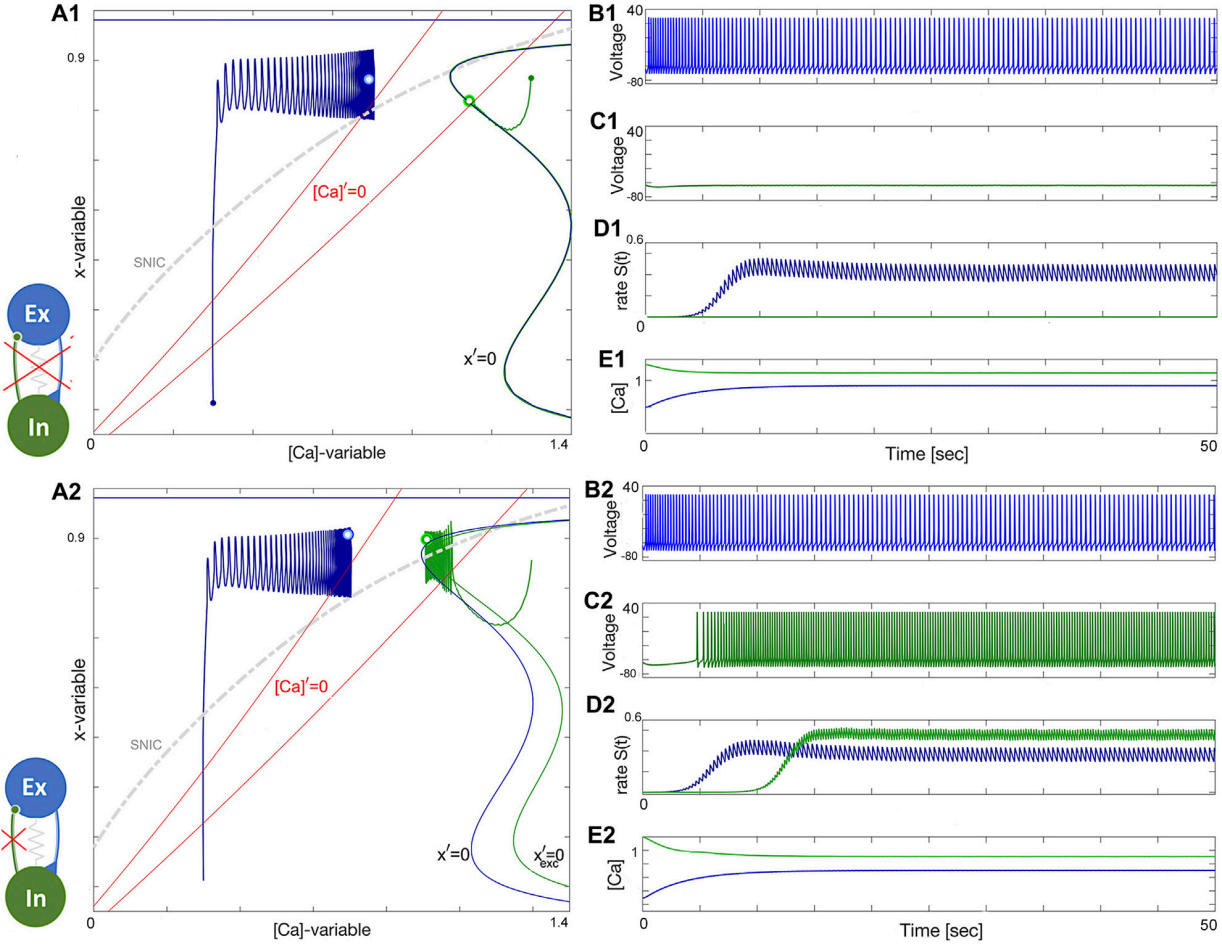
Building an E/I-module. Panels **(A1-E1)**: uncoupled excitatory tonic-spiking neuron (blue) at Δ_Ca_ =−50mV and inhibitory quiescent neuron (green) at Δ_Ca_ =−20mV. Panels **(A2-E2)**: Turning the excitatory synapse “on” in the blue neuron makes the inhibitory neuron spike tonically as well. **(A1, A2)** red lines labeled by [Ca]′=0 standing for the calcium nullclines above/below when the [Ca]-variable increases/decreases, the unperturbed and perturbed locations of the equilibrium states (double dots) are shown in the color-matching Σ-shaped nullclines *x*′=0 and $x_{\text{exc}}^{\prime }=0$ , as well as shows the position of the periodic orbits in the ([Ca], *x*)-phase planes for both neurons. The synaptic parameters are *g*_12_=0.2nS, *α*_12_=0.016, *β*_12_=0.001*s* ^−1^ and *g*_*elec*_ =0.0002nS and *g*_21_=0nS in the lower panels.

**FIGURE 15:**
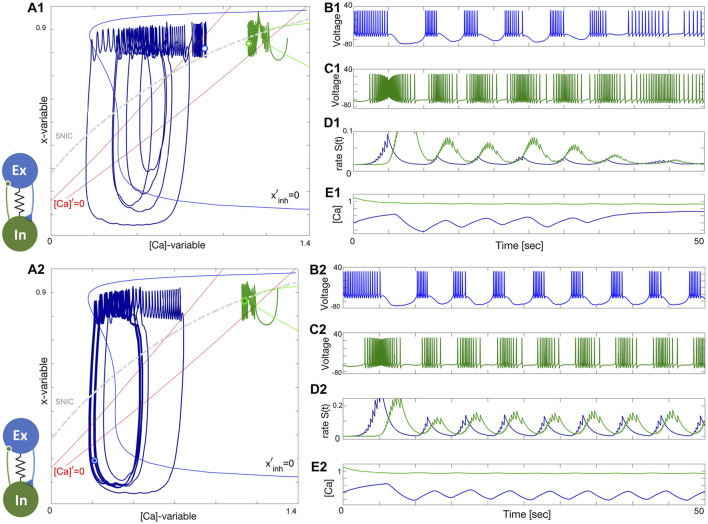
Building an E/I-module. Panels **(A1–E1)**: Turning the inhibitory synapse “on” makes the E/I-module initiate network bursting but cannot sustain due to a lack of balanced coupling so it breaks apart with its components returning to their natural states: a tonic-spiking neuron (blue) at Δ_Ca_ =−50mV and a quiescent neuron (green) at Δ_Ca_ =−20mV. Panels **(A2–E2)**: increasing inhibition lets the E/I-module sustain the network bursting stably as seen from the voltage and release rate *S*(*t*)-traces as well-seen from Panel **(B2-D2)**. Note both neurons alternate between tonic-spiking and quiescent phases demarcated by the SNIC-curve in the ([Ca], *x*)-phase plane in panels **(A1, A2)**. The synaptic parameters are *g*_12_=0.15, *α*_12_=0.0142, *β*_12_=0.001*s* ^−1^ and *g*_21_=0.2nS, and *α*_21_=0.01, *β*_21_=0.001*s* ^−1^ in the upper panels, and *g*_12_=0.2nS, *α*_12_=0.016, *β*_12_=0.001*s* ^−1^ and *g*_21_=0.5nS, *α*_21_=0.011, *β*_21_=0.001*s* ^−1^, and *g*_*elec*_ =0.0003*nS* in the lower panels.

The construction of the E/I module is detailed in Figures 14, 15. The network is silent with an uncoupled pair of one quiescent neuron and tonic neuron. As slow excitation is introduced the quiescent cell fires tonically after a delay. The introduction of inhibition shown in Figure 15 creates the conditions for bursting as the calcium dynamics in the excitatory cell and the slow excitation become resonant.

### 3.6 Tonic and quiescent neurons

Observe from the phase plane in Figure 15A2 that the inhibitory drive drives the excitatory (blue) neuron into a state of hyperpolarized quiescence along the approximate position of the forced equilibrium nullcline, $x_{\mathrm{inh}}^{\prime }=0$ . The inhibitory feedback becomes stronger with increasing excitatory drive, resulting in a longer recovery time for the excitatory (blue) neuron from its hyperpolarized state. This means that stronger coupling can prolong the interburst interval and the burst period of the network. However, increasing the strength of the bidirectional electric synapse (gap junction) allows the excitatory neuron to recover faster by counteracting the inhibitory effect and restoring the network balance, bringing the duty cycle of network bursting closer to the necessary phase relationship to sustain oscillations. This relationship is evident in the *S*(*t*)-traces represented in Figure 15D2.

Note that bursting oscillations in the voltage trace of the inhibitory neuron occur due to its cycling between a meta-stable state due to excitation drive and its natural resting state, which it receives no drive while the excitatory neurons transitions throughout forced hyperpolarized episodes. These cycling translates into small oscillations crossing the SNIC-curve in the ([Ca], *x*)-plane around [Ca] = 1.15 in Figure 15A2–E2. Unlike large-amplitude network limit cycle of the excitatory neuron, the oscillations corresponding to the inhibitory one are hardly noticeable. Nevertheless, as long as the transient stays above or below the SNIC-curve, the corresponding voltage traces will respond, respectively, with spike trains alternating with quiescent intervals, see Figure 15B2, C2.

**FIGURE 16:**
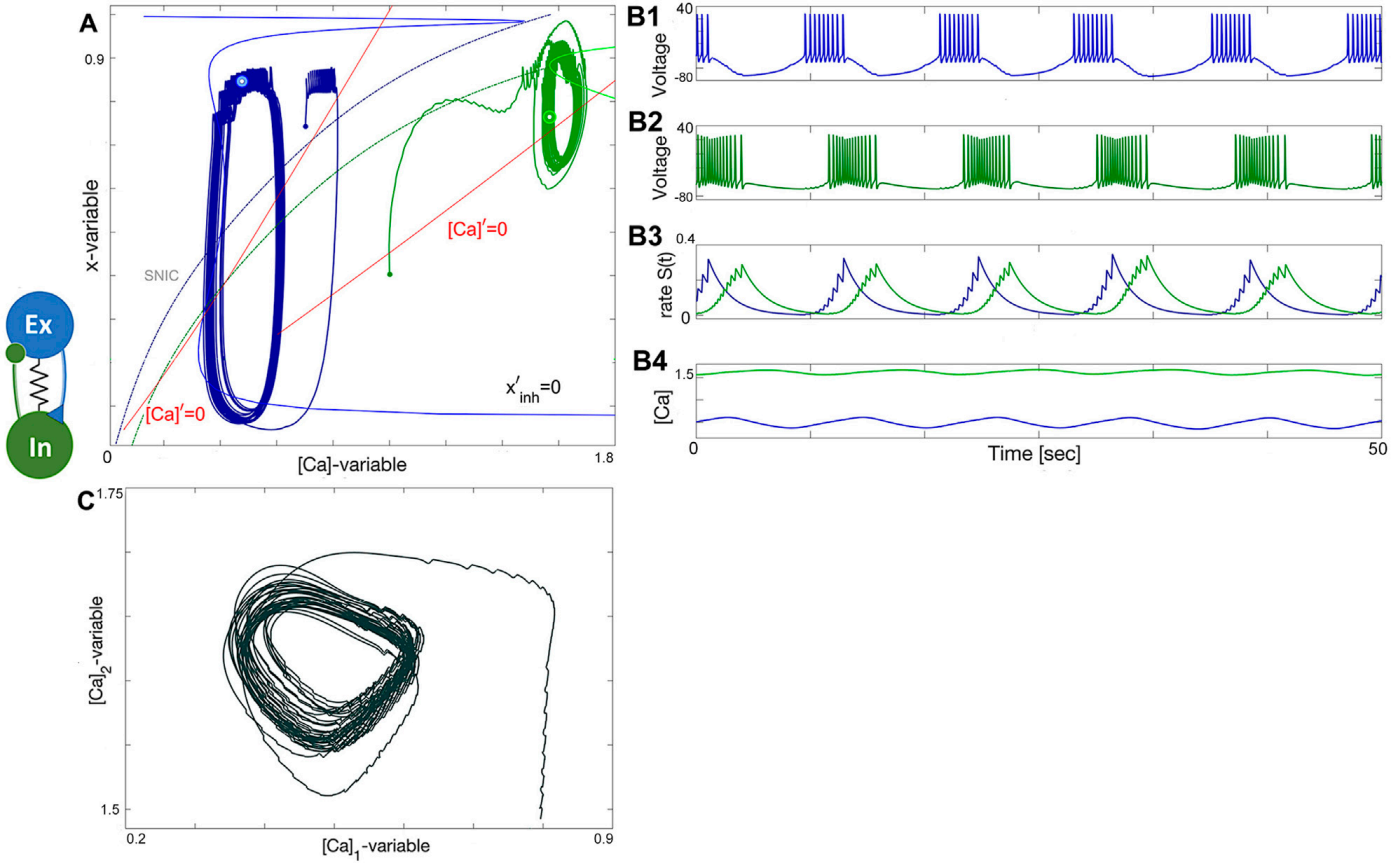
*Prey-predator network.* **(A)** diverse E/I-module constituted of a tonic-spiking neuron (blue) at Δ_Ca_ =−60mV and a quiescent neuron (green) at Δ_Ca_ =+60mV that reciprocally produce robust bursting in voltage traces **(B1–B4)** corresponding to a stable limit cycle in the ([Ca], *x*)-plane in Panel **(A)** and the $\left({\left[Ca\right]}_{1},\;{\left[Ca\right]}_{2}\right)$ -phase plane in Panel **(C)** due to both factors: sufficiently strong coupling and a phase-lag ∼1/4 period of oscillations between excitatory and inhibitory fluxes of neurotransmitter releases seen in Panel **(B3)**. Decreasing the inhibitory strength makes the oscillations less pronounced. Note two phase point snapshots, blue and green dots located at 12 o’clock (above) and nine o’clock (left) on the limit cycles lock positions on the two cycles in Panel **(A)** indicating a 1/4-period phase-lag between the oscillations in the excitatory and inhibitory neurons, resp. The synaptic parameters are *g*_12_=0.03 and *g*_21_=0.1nS, while *α*_12_=0.02, *β*_12_=0.0011, *α*_21_=0.012, *β*_21_=0.001 all in *s* ^−1^, and *g*_*elec*_ =0.001nS.

To demonstrate the stability and parameter range of network bursting, we make the excitatory and inhibitory neurons diverse. The excitatory neuron is set to have an active tonic-spiking behavior at Δ_Ca_ = −60 mV, while the inhibitory neuron is set to be deeply hyperpolarized at Δ_Ca_ = 20 mV, and undergoes the same assembly stages as before. The results are shown in Figure 16. The phase plane in Figure 16A shows that the inhibitory neuron has longer and deeper excursions between its two states: tonic-spiking and quiescence, as evidenced by the green cycle and the SNIC curve. The longer period of emergent bursting is also evident in the voltage traces (Figure 16B1, B2). In addition, Figure 16C shows a stable limit cycle in the $\left({\left[Ca\right]}_{1},{\left[Ca\right]}_{2}\right)$ -phase plane, indicating a proper balance of amplitudes and phases, with a phase-lag close to one quarter (1/4) period. This is confirmed by the instantaneous snapshot of the current phases of both neurons shown as two double dots (blue at 12 o’clock and green at nine o’clock) on the corresponding limit cycles in the ([Ca], *x*)-phase plane.

### 3.7 Two quiescent neurons to generate perpetual bursting

**FIGURE 17:**
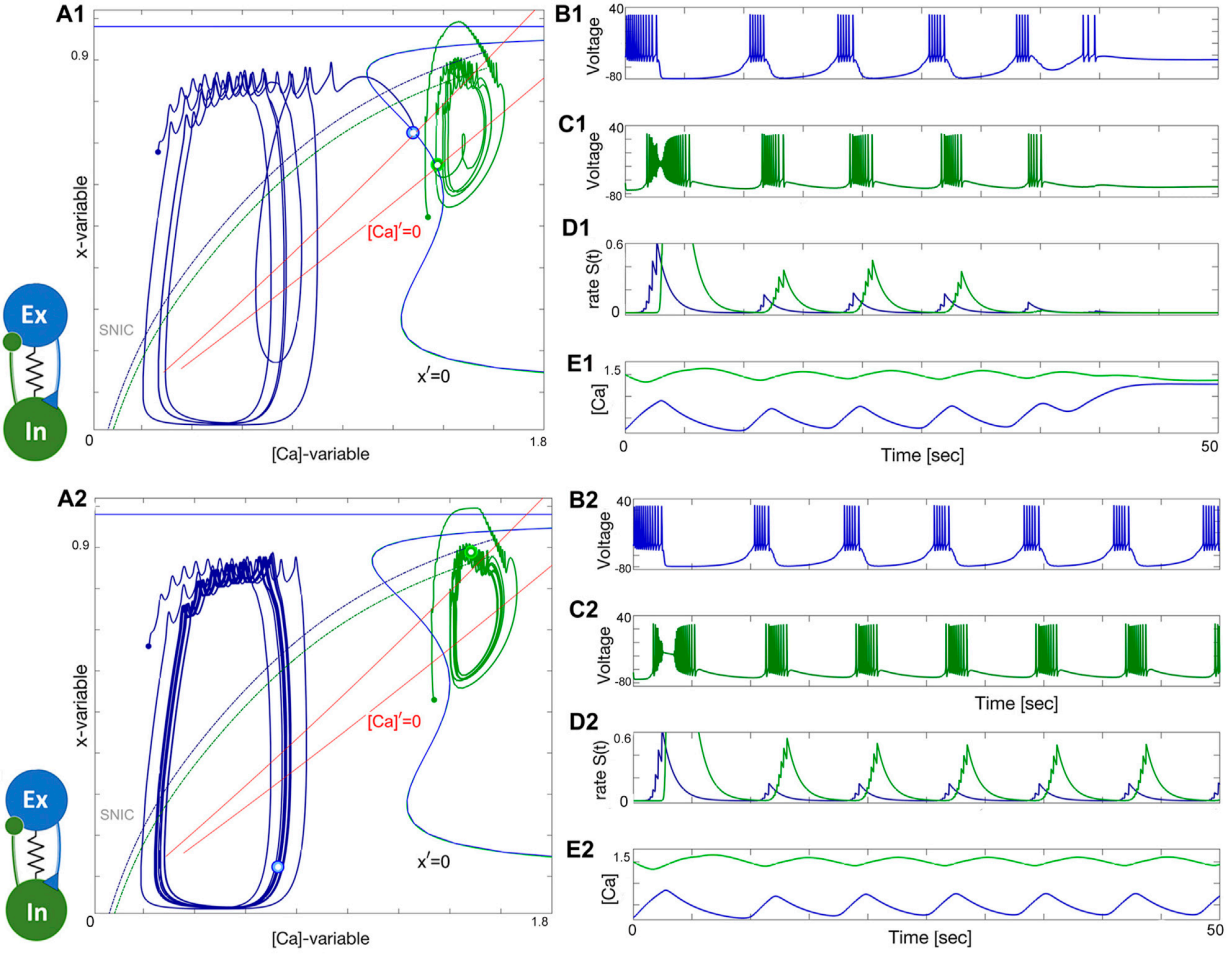
Panels **(A1-E1)**: The network bursting in the E/I-module loses its initial momentum and transitions back to the native quiescent states: the excitatory (blue) neuron at Δ_Ca_ =20mV and the inhibitory (green) neuron at Δ_Ca_ =60mV. **(A1)** Equilibrium states are shown as blue and green double dots at the intersection of the nullclines [Ca]′=0 and *x*′=0 in the ([Ca], *x*)-phase plane. Panels **(A2-E2)**: Increasing the forward excitation warrants self-sustained bursting oscillations with a phase-lag close to 1/4. Here, the synaptic parameters are *g*_*elec*_ =2.5×10^−4^nS, *α*_*ex*_ =0.033, *β*_*ex*_ =0.001, *α*_*in*_ =0.021, *β*_*in*_ =0.001 all in *s* ^−1^, *g*_*ex*_ =0.0375nS and 0.045, *g*_*in*_ =0.6nS.

Next, let us discuss two more options to generate emergent bursting in the EI-module. In the first case, both neurons are naturally quiescent at different Δ_Ca_-values, as can be deduced from their voltage traces in Figure 17B1, C1. This figure depicts that the excitatory neuron is initiated in the tonic-spiking phase far from its natural equilibrium (represented by the small blue dot) to produce a flux of positive feedback to the inhibitory neuron, which is initially set closer to its native equilibrium state (green dot). We can deduct from this figure that this EI-module is misbalanced and cannot keep up its initial momentum, as seen in the synaptic rate *S*(*t*) traces in Figure 17D1, which decay and slow down emerging bursting back to the quiescent states in both neurons. Those are represented by the blue and green double-dots through which the (red) nullclines [Ca]′ = 0, left for Δ_Ca_ = 20mV and right for Δ_Ca_ = 60mV, cross the Σ-shape nullcline *x*′ = 0 in the ([Ca], *x*)-projection in Figure 17A1.

Increasing the excitation remedies the situation, as one see from see Figure 17A2-E2. So, it appears that newly born stable limits cycles underwent through a network version of an Andronov-Hopf bifurcation in the slow phase plane in Figure 17A2 that gave rise to the onset of steady self-sustained bursting. This bursting is due to a strong or balanced level of reciprocal nonlinear interplay of excitatory and inhibitory currents flowing back and forth through feedback looks between the constituent neurons. Recall that while coupling can be sufficient for the occurrence of self-sustained bursting, it can fail at the initial stage because of an improper balance of the phases or a phase-lag between the neurons in the EI-module. On the other hand, even with proper initial phases, the network may not gain the initial momentum, and bursting will slow down and seize just like in the previous case (Figure 17A1). This is an indicator that likewise the HCO, the EI-module is bi-stable as well, and moreover network oscillations are composed of transient trajectories generated by its components. This is reminiscent to the concept and principles underlying perpetual motions observed in various pairs of diversely connected mechanical bodies (Angrist, 1968).

Let us reiterate that the phase-lag between oscillations generated by the advancing excitatory neuron and the following inhibitory neuron is to be close to a quarter of the network period. This is apparent in Figure 17A2 (as well as panel D2), which captures a snapshot of the relative positions of the blue and green phase points on the corresponding cycles at 5 (lower right) and 12 o’clock (above) respectively. Recall that the change rate of the gating *x*-variable is faster than that of [Ca]-dynamics, and therefore the phase points do not turn along the cycles uniformly (with non-constant orbital speeds). Therefore, such snapshots can show some variability of the phase lags between oscillations fluctuating around the target value of 1/4 on average.

### 3.8 Two tonic-spiking neurons

**FIGURE 18:**
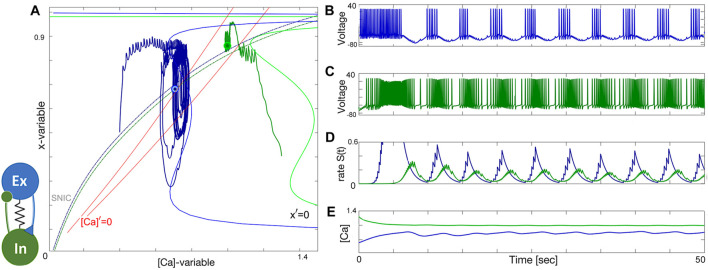
Inhibition-driven bursting in the excitatory (blue) neuron [its phase projection in **(A)**] reciprocally causes a slow frequency modulation in the voltage trace of the inhibitory (green) neuron in the E/I-module; here both neurons are set to spike tonically as shown in **(B–E)**, resp., at Δ_Ca_ =−60mV and Δ_Ca_ =−40mV; *g*_*elec*_ =4⋅10^−3^nS, while *α*_*ex*_ =0.025, *β*_*ex*_ =0.001, *α*_*in*_ =0.01, *β*_*in*_ =0.0014 all in *s* ^−1^, and *g*_*ex*_ =0.01nS and *g*_*in*_ =0.15nS.

The bistability and the conditions of balanced initial phases and mixed-coupling remain valid also in the case of the excitatory-inhibitory module comprised of two tonically spiking neurons at two distinct values Δ_Ca_ = −60mV and Δ_Ca_ = −40mV, see Figure 18. One can see from the voltage traces shown in Figures 18B, C that the blue excitatory neuron demonstrates bursting activity with quiescent phases caused by the flux of the inhibitory current generated by the green inhibitory one. The stronger the inhibition current is, the longer the excitatory neuron recovers from it. This feedback mechanism alone can regulate the duty cycle of network bursting in the EI-module. Note that the inhibition becomes stronger as the excitatory drive increases; the inhibitory neuron receives the excitatory drive and becomes even more *hyper* active with a greater spike frequency through the forward loop and *vice versa*. Because both neurons are initially chosen to demonstrate tonic-spiking activity, the voltage trace of the inhibitory one in Figure 18C demonstrates a spike frequency modulation caused periodically by the positive drive originating from the excitatory neuron. In turn, those cycling episodes of higher frequency cause stronger inhibition feedback looped back onto the excitatory neuron that warrants the quiescent interburst intervals in its traces, and so forth. At this point, the role of stronger electrical coupling between both neurons comes into play. As we mentioned earlier the electrical gap junction equates the dynamics of the coupled neurons, and even synchronizes them in the absence of mixed chemical synapses. In the given case, depending on the inhibitory/excitatory balance, increasing the electric coupling can produce two possible outcomes: i) depolarizing the excitatory neuron and shortening or illuminating interburst intervals and bringing it back to the original tonic-spiking activity. In the second case ii) inhibition “prevails” over excitation, so that the interburst episodes when the excitatory neuron becomes deeply hyperpolarized around −70 to−75mV, also bring down the voltage in the inhibitory neuron, and its spike frequency decreases substantially. One can observe fast EPSPs between bursts in the voltage trace (Figure 18B) of the excitatory neuron caused by the electrical current in a correlated response to spikes generated by the inhibitory one. This “equating” action by the gap junction can be also seen in the position of the corresponding cycles in the ([Ca], *x*)-projection in Figure 18A: weakening the strength of electrical coupling noticeably increases the amplitude of forced oscillations in the blue excitatory neuron. On the contrary, gradually increasing the electrical coupling will misbalance the excitatory/inhibitory ratio, eventually bringing both cycles closer in the phase plane, and narrowing the phase-lag between them will result in halting the burst rhythmogenesis in the excitatory-inhibitory module.

We wrap up the EI-module section with the following conclusions:

• emergent EI-bursting is a network-hysteresis based phenomenon, and stems from transient dynamics in both constituent neurons;
• EI-bursting bursting becomes self-sustained provided that both balances of initial phases and coupling strength are met;
• when either balance condition fails, network bursting disintegrates and the EI-neurons return to their natural states;
• EI-module is a bistable network;
• logistic synapses make emergent IE-bursting flexible, and less stiff compared to *α*-synapses;
• EI-module can be comprised of tonic spiking and quiescent neurons, and both tonic-spiking neurons, which warrants its broad structural stability range;
• phase-lag in EI-bursting can fluctuate between $\frac{1}{4}$ and $\frac{1}{2}$ as the inhibitory synapse becomes more delayed.

## 4 Discussion

The understanding of CPGs can be improved by identifying the right dynamic constructions and their characteristics, especially regarding the output behavior’s flexibility and stability in response to parameters. Although single neuron models and simple patterns have been studied, more research is needed to explain how small network features lead to adaptable behavior. The versatility of two CPG networks in this paper is demonstrated by their gradual convergence to the bursting attractor and their easy control by cellular and synaptic parameters. Further formalizing the dynamic principles and characteristics of these subnetworks will expand the network motifs and adaptability mechanisms.

This paper highlights two key concepts. Firstly, a more accurate method is required to determine and measure the dynamic requirements for oscillatory activity in neural circuits beyond the motifs illustrated in this paper. To address this, the paper introduces the idea of network hysteresis, which stems from the discovery that in self-oscillating neural systems where cells do not have active hysteresis, hysteresis must be shared between cellular and synaptic variables. Further research will delve into methods for identifying and analyzing the subsystems and manifolds involved in hysteretic activity across networks.

The second objective of this paper is to offer versatile building blocks for the CPG circuits in sea slug swimming. In future studies, these motifs will be used to construct and analyze larger CPG networks. The parameters that work best for these large CPGs may not match precisely with the parameters for bursting, but we still anticipate that the sequence of transients that defines the bursting in the small networks we present here will bear enough resemblance to the mechanisms in larger circuits to be helpful in explaining them.

This paper offers a bifurcation analysis of the swim interneuron model presented here but does not examine chaotic transitions in detail. A full dynamical analysis of the swim interneuron model is left to future research.

## 5 Conclusion

In conclusion, this paper provides a comprehensive overview of two mechanisms of rhythm generation in pair-wise networks. The first mechanism, emergent bursting in a reciprocally inhibitory network, has been shown to be capable of generating flexible rhythms over a wide range of initial states through the coordination of transients driven by synaptic current. The second mechanism, an excitatory/inhibitory pair, demonstrates the stability and multistability of different configurations that correspond to the endogenous behavior of the cells.

Moreover, the paper presents a conductance-based model for swim CPG interneurons in sea slugs *Melibe leonina* and *Dendronotus iris*. The Plant model (Plant and Kim, 1975; Plant and Kim, 1976; Plant, 1981) was selected as the starting point for the developed SiN-model due to its well-known variability in spike frequency. However, the original one was modified to eliminate bursting, which is not observed in swim interneurons, by introducing two additional bifurcation parameters and an *h*-current, as well as using an averaging approach for fast-slow decomposition. The effect of these modifications, as well as the effect of synaptic drive, was shown to provide a better understanding of the behavior of these neurons in a network setting. Additionally, a novel synapse called the logistic synapse was introduced, which can effectively act as a high pass filter and create a delay.

The neural circuits discussed in this paper overlap to form the CPG networks in *Melibe leonina* and *Dendronotus iris*. This work serves as a foundation for future studies to explore the interaction of these two mechanisms and the properties of the resulting system in the context of animal locomotion.

## Data Availability

The toolkit that supports the findings of this study is openly available and deposited in GitHub at https://github.com/jamesjscully/plant_paper. Our software codes including equations, parameters etc. are available upon request.

## Footnotes

1 The Plant model generates bursts with deeply hyperpolarized quiescent sags, see Figure 4B. To flatten/rectify those sags, the *h*-current was additionally introduced in the interneuron model, and we would like to thank to D. Terman for the suggestion. This current also prevents unrealistic levels of hyperpolarization in response to negative external currents applied to the neuron model. The use of *h*-current, though practical, does not appear to play a critical role in the swim CPG models.

2 N.N. Bautin examined a structure of such a point where the criticality of this bifurcation alters from sub to super, and found that its unfolding includes an additional curve corresponding to a saddle-node bifurcation of periodic orbits that originates off the codimension-2 point (BP). Note that the occurrence of such a point in various neuronal models is a precursor of a possible transition to bursting through a torus bifurcation (Ju et al., 2018).

3 SNIC (Saddle-Node-on-Invariant-Circle) stands for a homoclinic bifurcation of a saddle-node periodic orbit of codimension-one, which was first examined by Andronov and Leontovich (Andronov and Leontovich, 1937; Andronov et al., 1938; Andronov et al., 1967) in a plane, and further generalized by L.P. Shilnikov for the **R** ^*n*^-case in his Ph.D. thesis, see Refs (Shilnikov, 1962; Shilnikov, 1963; Shilnikov et al., 1998; Afraimovich et al., 2014; Afraimovich et al., 2017; Gonchenko et al., 2022). and references therein. The feature of a SNIC bifurcation is that it unfolds with the onset of either a stable periodic orbit of a long period emerging from a homoclinic orbit to a saddle-node, or a stable equilibrium.
