# Supplementary material for: Pairing cellular and synaptic dynamics into building blocks of rhythmic neural circuits. A tutorial
Source: Front Netw Physiol. 2024 Jun 25;4:1397151. doi: 10.3389/fnetp.2024.1397151 (PMC11231435; doi:10.3389/fnetp.2024.1397151)
Supplement: Supplementary file 1 [file DataSheet1.pdf]

APPENDIX I: A BASIC HODGKIN-HUXLEY BASED MODEL OF THE SWIM CPG INTERNEURONS

The dynamics of the membrane potential, V, is governed by the following equation:

$$C_m V' = -I_I - I_K - I_T - I_{KCa} - I_h - I_{leak} - I_{syn}. \tag{1}$$

The fast inward sodium and calcium I_I-current is given by

$$I_I = g_I h m_\infty^3(V)(V - E_I), \tag{2}$$

with the reversal potential E_I = 30mV and the maximal conductance value g_I = 4nS, and

$$m_\infty(V) = \frac{\alpha_m(V)}{\alpha_m(V) + \beta_m(V)}, \tag{3}$$

$$\alpha_m(V) = 0.1 \frac{50 - V_s}{-1 + e^{(50 - V_s)/10}}, \tag{4}$$

$$\beta_m(V) = 4e^{(25 - V_s)/18} \tag{5}$$

the dynamics of its inactivation gating variable h is given by

$$h' = \frac{h_\infty(V) - h}{\tau_h(V)}, \quad \text{where} \tag{6}$$

$$h_\infty(V) = \frac{\alpha_h(V)}{\alpha_h(V) + \beta_h(V)} \quad \text{and} \tag{7}$$

$$\tau_h(V) = \frac{12.5}{\alpha_h(V) + \beta_h(V)} \tag{8}$$

$$\alpha_h(V) = 0.07e^{(25 - V_s)/20}, \tag{9}$$

$$\beta_h(V) = \frac{1}{1 + e^{(55 - V_s)/10}}, \quad \text{and} \tag{10}$$

$$V_s = \frac{127V + 8265}{105} \text{mV}. \tag{11}$$

The fast potassium I_K-current is given by the equation

$$I_K = g_K n^4 (V - E_K), \tag{12}$$

with the reversal potential $E_K = -75$mV and the maximal conductance set as $g_K = 0.3$nS. The dynamics of inactivation gating variable are described by

$$n' = \frac{n_\infty(V) - n}{\tau_n(V)}, \quad \text{with} \tag{13}$$

$$n_\infty(V) = \frac{\alpha_n(V)}{\alpha_n(V) + \beta_n(V)} \quad \text{and} \tag{14}$$

$$\tau_n(V) = \frac{12.5}{\alpha_n(V) + \beta_n(V)}, \quad \text{where} \tag{15}$$

$$\alpha_n(V) = 0.01 \frac{55 - V_s}{e^{(55-V_s)/10} - 1} \tag{16}$$

$$\beta_n(V) = 0.125 e^{(45-V_s)/80}. \tag{17}$$

The rapidly depolarizing h-current is given by

$$I_h = g_h \frac{y(V - E_h)}{(1 + e^{-(V-63)/7.8)})^3}, \tag{18}$$

with $E_h = 70$mV, and $g_h = 0.0006$nS; the dynamics of its y-activation probability is described by

$$y' = \frac{1}{2}\left[\frac{1}{1 + e^{10(V-50)}} - y\right] / \left[7.1 + \frac{10.4}{1 + e^{(V+68)/2.2}}\right], \tag{19}$$

whereas the leak current is given by

$$I_{leak} = g_L(V - E_L), \tag{20}$$

with $E_L = -40$mV, and $g_L = 0.003$nS. The sub-group of slow currents in the model includes the TTX-resistant sodium and calcium I_T-current given by

$$I_T = g_T x(V - E_I), \tag{21}$$

with $E_I = 30$mV and $g_T = 0.01$nS, where the dynamics of its slow activation variable are described by

$$x' = \frac{x_\infty(V) - x}{\tau_x}, \quad \text{where} \tag{22}$$

$$x_\infty = \frac{1}{1 + e^{-0.15(V+50-\Delta_x)}}, \tag{23}$$

with the time constant τ_x set as 100 or 235ms in this study. The slowest outward Ca^{2+} activated K^+ current given by

$$I_{KCa} = g_{KCa} \frac{[Ca]_i}{0.5 + [Ca]_i}(V - E_K) \tag{24}$$

with $E_K = -75$mV, and $g_{KCa} = 0.03$, where the dynamics of intracellular calcium concentration is governed by

$$[Ca]' = \rho\left(K_c x(E_{Ca} - V + \Delta_{Ca}) - [Ca]\right) \tag{25}$$

with the Nernst reversal potential E_{Ca} = 140mV, and small constants ρ = 0.0003ms^{-1} and K_c = 0.0085mV^{-1}.

APPENDIX II: IMPLEMENTATION DETAILS FOR FAST-SLOW DECOMPOSITION

In this appendix, we describe the computational approaches used to determine the relative positions of the slow nullclines, the SNIC bifurcation curve, and the average quantities needed to locate periodic orbits, as well as to create the heat maps used in the figures of the main text.

Approach I: Equations of slow nullclines

To locate the positions of the slow nullclines $x' = 0$ and $[\mathrm{Ca}]' = 0$ in the $([\mathrm{Ca}]\,\mathrm{x})$-phase plane in the figures above, we first parametrize the membrane voltage V_n within an equilibrium range $[-70; +20]$mV with some step size. Next, solve the equilibrium state equation $V' = 0$ for the I_{KCa}-current:

$$I_{KCa} = -I_I - I_K - I_T - I_h - I_{leak} - I_{syn},$$

with the corresponding static functions $h_\infty(V_n)$, $n_\infty(V_n)$, and $m_\infty(V_n)$ for equilibria in the currents above, and next using Eq. (24) find the parameterized array

$$[\mathrm{Ca}(V_n)] = 0.5\,\frac{I_{KCa}}{g_{KCa}(V_n - E_k) - I_{KCa}}.$$

The ordered pairs $\{[\mathrm{Ca}(V_n)], x_\infty(V_n)\}$ populate the sought x-nullcline $x' = 0$. To determine the position of the nullcline $[\mathrm{Ca}]' = 0$, first solve the equation $V' = 0$ for the I_T-current:

$$I_T = -I_I - I_K - I_{KCa} - I_h - I_{leak} - I_{syn},$$

next find

$$x(V_n) = \frac{I_T}{g_T(E_I - V_N)},$$

and then from Eq. (25) find

$$[\mathrm{Ca}(V_n)] = g_T\, x(V_n)\,(E_{Ca} - V_n + \Delta_{\mathrm{Ca}}),$$

and use the ordered pairs $\{[\mathrm{Ca}(V_n)], x_\infty(V_n)\}$ to locate the calcium nullcline $[\mathrm{Ca}]' = 0$ in the slow $([\mathrm{Ca}]\,\mathrm{x})$-phase plane.

Approach II: Averaging approach to identify periodic orbits

To appraise and quantify the fast spiking behavior of the model via its fast subsystem, we need to determine the location of a corresponding stable orbit on the M_{po}-manifold and its 2D-projection, as depicted in Figs. 4A and B, respectively. Following Refs.[62, 81, 82], one numerically solves the following averaged system adopted from Eqs. (10)-(11) for a pair $(\langle x\rangle^*, \langle[\mathrm{Ca}]\rangle^*)$:

$$\langle x\rangle^* = \frac{1}{T}\int_0^T \frac{dt}{1 + e^{-0.15(V_{\mathrm{po}}(t)+50-\Delta_x)}}, \tag{26}$$

$$\langle[\mathrm{Ca}]\rangle^* = K_c E_{Ca} \langle x\rangle^{+} \Delta_{Ca} - \frac{K_c}{T}\int_0^T x_{\mathrm{po}}(t)\, V_{\mathrm{po}}(t)\, dt, \tag{27}$$

where $V_{po}(t)$, $x_{po}(t)$, and $[Ca_{po}(t)]$ are the coordinates of the periodic orbit with period T, situated on the critical 2D M_{po}-manifold at the given (Δ_{Ca}, Δ_x)-parameter values. Here $\langle x \rangle$ is the average quantity from Eq. (12). The solution $(\langle x \rangle^*, \langle [Ca] \rangle^*)$ of the average system above corresponds to "gravity center"of the sought periodic orbit in the phase pace of the swim interneuron model.

In practice, there is a (computationally) cheap trick for finding a loose approximation of the location and the gravity center of such a tonic spiking orbit in the phase space of the swim interneuron model. By setting $\tau_x = 1000$s or larger values one makes the dynamic x-variable slow so that it cannot echo fast voltage oscillations, but traces out the sought average $\langle x \rangle$ nullcline in the $([Ca], x)$-phase plane. Its intersection with the calcium nullcline $[Ca]' = 0$ pin-points a location of the sought periodic orbit on the tonic-spiking manifold M_{po}.

Bi-parametric sweep of neuronal dynamics

The second approach employs contour lines identified in bi-parametric sweeps of the fast subsystem, where the x and $[Ca]$ variables become frozen to be treated as control parameters in the fast subsystem of the swim interneuron model. First, we create a grid (of $[300] \times [300]$-resolution) of ordered ([Ca], x)-pairs over the slow phase plane. For each pair, a phase trajectory of the model is computed and the initial transient is discarded. The trajectory is classified as either a spiker if the voltage has three or more zero crossings, and quiescent otherwise. Each case is treated separately but in both cases, four quantities are characterized: spike frequency, and average values $\langle V \rangle$, $\langle x \rangle$ and $\langle [Ca] \rangle$. When the system is quiescent, the frequency is 0, the average voltage is the final voltage, which is simply plugged into the x and $[Ca]$ state equations to determine $\langle x \rangle$ and $\langle [Ca] \rangle$-values.

In the case of tonic-spiking activity, we use the final three zero crossings of the entire trajectory to determine the established spike frequency and the average voltage. The x- and $[Ca]$-coordinates are calculated for every point in the sampling of the limit cycle and subsequently averaged. This order of operations is required due to nonlinearities in the model dynamics. The nullclines are plotted as the zero contours of the resulting $\langle x \rangle$ and $\langle [Ca] \rangle$ arrays, while the SNIC-curve itself is evaluated as the zero contours of the frequency array. The average voltage is only used to generate the corresponding heat-map color-painting in the phase plane with a red hue for its oscillatory part above the SNIC-curve, and below it with a blue hue representing quiescent phases.

APPENDIX III: SEQUENTIAL PHASE PLANE ANALYSIS OF NETWORK PERIODIC ORBITS

In order to validate the mechanism underlying rhythmogenesis in the two network models we visualize the movement of the nullclines over the course of a network bursting cycle. We partitioned a network bursting trajectory into subsets where all $S(t)$ were monotonic in time and visualized the transformations of the geometry as the network oscillations unfolded. During our investigation of the excitatory-inhibitory module, we noticed that it was also possible to obtain network oscillations with frozen x and $[Ca]$-variables, suggesting that the mechanism of rhythmogenesis was not a product of the slow phase dynamics alone. Further investigation into the excitatory-inhibitory module is required.

We showed above that in the case of the HCO network, the onset of network oscillations emerging from voltage transients is coordinated by the cyclicly moving x-nullclines $x' = 0$ in the slow ([Ca], x)-phase plane for both neurons. Here, we include a supplementary Fig. 1 to further detail and validate our analysis. Its panels A and D for neurons 1 and 2, respectively, demonstrate representative snapshots of time-varying

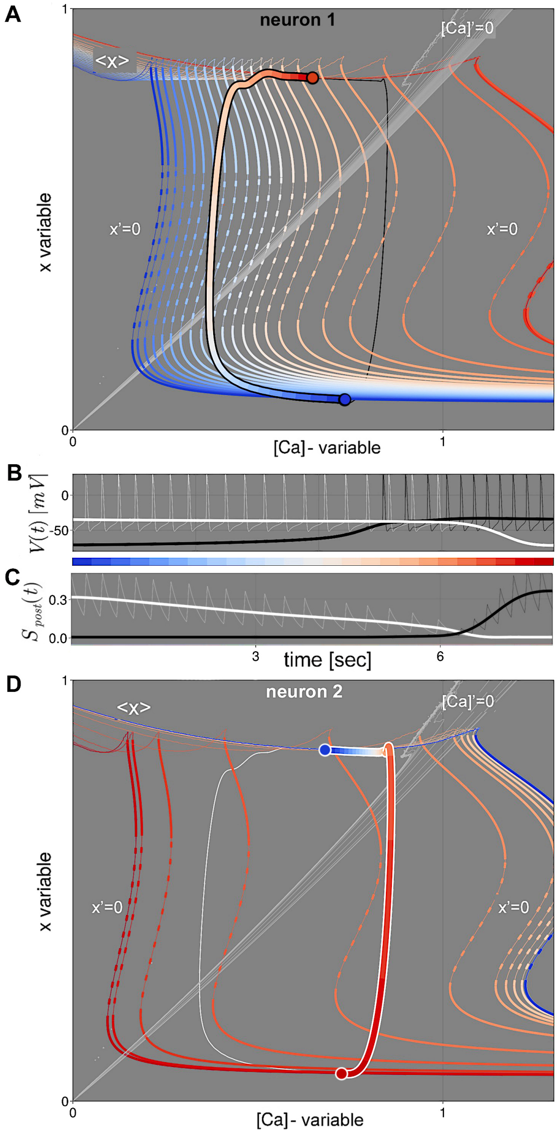

Figure 1. Illustration of the dynamic evolution of the slow nullclines, equilibrium $x' = 0$, average $\langle x \rangle$, and calcium $[\mathrm{Ca}]' = 0$, in the slow $[\mathrm{Ca}, \mathrm{x}]$-phase plane overlaid with the thick semicircular bursting orbits for both neurons in the HCO network over its half period. In all panels, the color spectrum, varying from the blue to the red color, is meant to communicate time progression, whereas black and white lines are associated with neurons 1 and 2, resp. The thick lines in Panel B represent the smoothed the voltage traces $V(t)$, whereas the thin one represent the original ones. The time progressions of the synaptic variables $S_{post}(t)$ for both neurons are presented in Panel C. Panels A and D demonstrate small deviations of the calcium nullcline $[\mathrm{Ca}]' = 0$ over a burst cycle, while the perturbed equilibrium nullclines $x' = 0$ in the post-synaptic neurons are shifted by the inhibition far leftwards in the phase plane compared with unperturbed ones on the right in the pre-synaptic neurons.

rearrangements of the nullclines, equilibrium $x' = 0$, average $\langle x \rangle$ and calcium $[\mathrm{Ca}]' = 0$, in a chronological sequence over a half of each burst cycle. Since the HCO network is symmetric, the neurons will next switch their roles to complete the full bursting cycle. Close examination yields an interpretation by breaking a

half-cycle sequence into two stages:
Stage I. As the active pre-synaptic neuron 2 (its initial/final positions given by blue/red dots in Panel D) drifts rightwards and approaches the SNIC-curve (not shown) along the average $\langle x \rangle$ nullcline, its spike frequency decreases so that the synaptic probability $S_2(t)$ (black line in Panel C) drops to zero. The concurrent decay of inhibition projected from neuron 2 onto neuron 1 (its initial/final positions given by blue/red dots in Panel A) lets the corresponding x_1-variable rise, which in turn makes the average $\langle x \rangle$ nullcline for neuron 2 shift to the right.
Stage II. After the x_1-variable reaches the average $\langle x \rangle$ nullcline (color-coded red line in Panel A), neuron 1 starts spiking and hence inhibiting neuron 2. The inhibition causes the average $\langle x \rangle$ nullcline for neuron 2 to shift leftwards so that the x_2 variable drop down close to zero and that the corresponding phase trajectory of neuron 2 is about to start traversing along the forced (red line) equilibrium nullcline $x' = 0$ in the phase plane in Fig. 1D, and so forth.

To smooth a $V(t)$ trace of the active spiking neurons, it is first partitioned to ensure that each spike belongs to a unique subset by monitoring a threshold set around -35mV. A maximum spike time of 1s determines whether each subset contains a spike or a quiescent episode of the burst. Spike-containing subsets are replaced with their voltage averages, while quiescent voltage level are left intact. The partitioning and averaging for each $S(t)$ follow their corresponding presynaptic voltages. Finally, a composition of two moving averages with a 0.5s window is applied as a filter for further smothering.
